# Structured Demographic Buffering: A Framework to Explore the Environmental Components and Demographic Mechanisms Underlying Demographic Buffering

**DOI:** 10.1111/ele.70066

**Published:** 2025-02-25

**Authors:** Samuel J. L. Gascoigne, Maja Kajin, Shripad Tuljapurkar, Gabriel Silva Santos, Aldo Compagnoni, Ulrich K. Steiner, Anna C. Vinton, Harman Jaggi, Irem Sepil, Roberto Salguero‐Gómez

**Affiliations:** ^1^ Department of Biology University of Oxford Oxford UK; ^2^ School of Biological Sciences University of Aberdeen Aberdeen UK; ^3^ Department of Biology, Biotechnical Faculty University of Ljubljana Ljubljana Slovenia; ^4^ Biology Department Stanford University Stanford California USA; ^5^ National Institute of the Atlantic Forest (INMA) Santa Teresa Espírito Santo Brazil; ^6^ Institute of Biology Martin Luther University Halle‐Wittenburg Halle (Saale) Germany; ^7^ German Centre for Integrative Biodiversity Research (iDiv) Halle‐Jena‐Leipzig Leipzig Germany; ^8^ Institute of Biology Freie Universität Berlin Berlin Germany; ^9^ National Laboratory for Grassland & Agro‐Ecosystems Lanzhou University Lanzhou China

**Keywords:** environmental variability, integral projection models (IPMs), life history strategies, stochastic demography

## Abstract

Environmental stochasticity is a key determinant of population viability. Decades of work exploring how environmental stochasticity influences population dynamics have highlighted the ability of some natural populations to limit the negative effects of environmental stochasticity, one of the strategies being demographic buffering. Whilst various methods exist to quantify demographic buffering, we still do not know which environmental components and demographic mechanisms are most responsible for the demographic buffering observed in natural populations. Here, we introduce a framework to explore the relative impacts of environmental components (i.e., temporal autocorrelation and variance in demographic rates) on demographic buffering and the demographic mechanisms that underly these impacts (i.e., population structure and demographic rates). Using integral projection models, we show how demographic buffering is more sensitive to environmental variance relative to environmental autocorrelation. In addition, environmental autocorrelation and variance impact demographic buffering through distinct demographic mechanisms—i.e., population structure and demographic rates, respectively.

## Graphical abstract

Demographic buffering quantifies the degree to which a population's demographic rates reduce the negative impacts of environmental stochasticity. Here, we show that temporal variance and autocorrelation impact demographic buffering through different mechanisms.

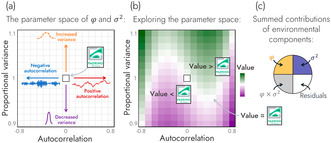

## 1 Introduction

Understanding how populations minimise the negative effects of environmental stochasticity is central to ecology and evolution (Sutherland et al. 2013). A key prediction of population ecology is that increased environmental stochasticity, defined as the temporal variation in demographic rates (e.g., rates of progression, stasis, retrogression and fertility) (Engen, Bakke, and Islam 1998), reduces a population's stochastic growth rate ($\lambda_{s}$) (Tuljapurkar 1982, 1989). In extreme cases, this variance in demographic rates can lead to local extinction (Bull et al. 2007; Lennartsson and Oostermeijer 2001; May 1973; Melbourne and Hastings 2008; Saether, Islam, and Perrins 1998). Environmental variability, hereon defined as the temporal variation in climate variables across timesteps (e.g., temperature, precipitation), is a key driver of variance in demographic rates (Jongejans et al. 2010). Importantly, the stochasticity in vital rates of species sensitive to environmental variables is projected to change across the globe due to climate change (e.g., temperature: Bathiany et al. 2018; Di Cecco and Gouhier 2018; Lewis and King 2017; Masson‐Delmotte et al. 2021; Shen et al. 2011; Urban 2015). Therefore, understanding the environmental components (e.g., temporal autocorrelation and variance in demographic rates) and demographic mechanisms influencing the relationship between environmental stochasticity and population dynamics is both important and timely.

Three key considerations are needed to relate demographic rate variance to population dynamics. First, increased demographic rate variance negatively effects a population's growth rate (Arthreya and Karlin 1971; May 1973). Second, the negative effects of demographic rate variance on population growth are exacerbated when variance occurs in the demographic rate(s) of highest importance (i.e., sensitivity) to $\lambda_{s}$. However, the negative effect of demographic rate variance on $\lambda_{s}$ can be reduced (or increased) when demographic rates covary negatively (or positively) (Tuljapurkar 1982, 1989), as demographic rates can compensate (amplify) for one another within a timestep (Sheth and Angert 2018). Third, the effects of environmental variance and variance in demographic rates on $\lambda_{s}$ can be uncoupled through curvilinear environment–demographic rate reaction norms (Bruijning et al. 2020; King and Hadfield 2019). Following Jensen's inequality (Jensen 1906), convex (∪‐shaped) environment–demographic rate reaction norms can result in a positive effect of environmental variance on $\lambda_{s}$ through increases in mean demographic rates, whereas concave (∩‐shaped) reaction norms lead to a negative effect through decreases in mean demographic rates (Drake 2005; Koons et al. 2009). These three key considerations regarding the impact of stochastic environments on population dynamics have produced key predictions in life history theory (Sæther et al. 2013; Tuljapurkar, Gaillard, and Coulson 2009), conservation biology (Foley 1994; Higgins, Pickett, and Bond 2000) and agriculture science (Lande, Sæther, and Engen 1997; Mack 2000). However, these three considerations alone do not allow us to quantify a population's ability to accommodate demographic rate variance; demographic buffering does.

Researchers have quantified demographic buffering primarily using two methods: A regression‐based approach and stochastic elasticities of variance. The regression‐based approach measures demographic buffering by regressing the deterministic—i.e., derived from the arithmetic mean of demographic rates across the timeseries—elasticities (or sensitivities) of population growth rate ($\lambda_{1}$) with respect to demographic rates against the temporal coefficient of variation (or variance) of demographic rates (Hilde et al. 2020; Morris and Doak 2004; Pfister 1998). The foundations for this approach lie in Tuljapurkar's approximation for stochastic growth rate (Tuljapurkar 1982, 1989, 1990):

(1) $$\log\left(\lambda_{s}\right)\approx \log\left(\lambda_{1}\right)-\frac{1}{2}\;\left[\sum_{\mathrm{ij}}e_{\mathrm{ij}}^{2}{\mathrm{CV}}_{\mathrm{ij}}^{2}+\sum_{\mathrm{ij}\neq \mathrm{kl}}e_{\mathrm{ij}}e_{\mathrm{kl}}\left(\frac{\mathrm{cov}\left(a_{\mathrm{ij}},a_{\mathrm{kl}}\right)}{\bar{a_{\mathrm{ij}}}\;\bar{a_{\mathrm{kl}}}}\right)\right]$$

Here, the difference between the logged values of $\lambda_{s}$ and $\lambda_{1}$ can be approximated by the summed products of demographic rate elasticities ($e_{\mathrm{ij}}$) and coefficients of variation (${\mathrm{CV}}_{\mathrm{ij}}$) values (the first term in the hard brackets) and their covariance structure (the second term in the hard brackets). Therefore, one method to minimise the impact of environmental stochasticity is through a negative covariance between $e_{\mathrm{ij}}$ and ${\mathrm{CV}}_{\mathrm{ij}}$ values—which the regression‐based approach specifically measures. The regression‐based approach has been broadly implemented for individual species (reviewed in Hilde et al. 2020) and comparative studies (McDonald et al. 2017; Pfister 1998). However, this approach has two key limitations. First, the covariance structure of $e_{\mathrm{ij}}$ and ${\mathrm{CV}}_{\mathrm{ij}}$ is not a term within Tuljapurkar's approximation relating the impact of environmental stochasticity on $\lambda_{s}$. And second, this method relies on the deterministic values of demographic rate importance ($e_{\mathrm{ij}}$) rather than the importance measure informed by demographic rate stochasticity.

Demographic buffering can also be quantified using the summation of stochastic elasticities of variance, $\sum E_{a_{\mathrm{ij}}}^{\sigma^{2}}$ (Santos et al. 2023; Wang et al. 2023). In this, $\sum E_{a_{\mathrm{ij}}}^{\sigma^{2}}$ quantifies the proportional contribution of demographic rate variance on $\lambda_{s}$ (Haridas and Tuljapurkar 2005; Tuljapurkar, Horvitz, and Pascarella 2003). In practice, $\sum E_{a_{\mathrm{ij}}}^{\sigma^{2}}$ is always negative. The reason $\sum E_{a_{\mathrm{ij}}}^{\sigma^{2}}$ is negative stems from the impact of environmental stochasticity always reducing $\lambda_{s}$ relative to $\lambda_{1}$. Importantly, Tuljapurkar's approximation can be written in terms of $\sum E_{a_{\mathrm{ij}}}^{\sigma^{2}}$ (Haridas and Tuljapurkar 2005):

(2) $$\log\left(\lambda_{s}\right)\approx \log\left(\lambda_{1}\right)+\frac{1}{2}\;\left[\sum E_{a_{\mathrm{ij}}}^{\sigma^{2}}\right]$$

This relationship between $\sum E_{a_{\mathrm{ij}}}^{\sigma^{2}}$ and Tuljapurkar's approximation makes $\sum E_{a_{\mathrm{ij}}}^{\sigma^{2}}$ a tractable measure of demographic buffering for questions of stochastic (Gascoigne et al. 2024; Westerband and Horvitz 2017) and comparative demography (Morris et al. 2008). Yet, whilst researchers have previously used $\sum E_{a_{\mathrm{ij}}}^{\sigma^{2}}$ to quantify demographic buffering (Dalgleish, Koons, and Adler 2010; Morris et al. 2008), we still do not know how different environmental components (i.e., temporal autocorrelation and variance), population structure (i.e., distribution of individuals in a population according to states, such as age, stage and/or size) and different demographic rates (i.e., state‐specific transition probabilities or reproductive contributions between time t and t+1) impact $\sum E_{a_{\mathrm{ij}}}^{\sigma^{2}}$. This represents a key gap in knowledge in our understanding of how demographic buffering capacities may shift in a changing world.

Here, we test the effects of environmental components, population structure and demographic rates on the ability of natural populations to remain demographically buffered. We use environmentally explicit, parameter‐stochastic integral projection models (IPMs) (Easterling, Ellner, and Dixon 2000; Ellner, Childs, and Rees 2016) for three perennial plant species from the PADRINO database (Levin et al. 2022) to test two hypotheses. We expect that: (H1) environmental autocorrelation and variance will have negative effects on $\sum E_{a_{\mathrm{ij}}}^{\sigma^{2}}$. Specifically, as environments become more variable and positively autocorrelated, populations will become less buffered, as predicted by Tuljapurkar's approximation (Tuljapurkar 1982, 1989, 1990). (H2) Environmental autocorrelation and variance influence $\sum E_{a_{\mathrm{ij}}}^{\sigma^{2}}$ via different demographic mechanisms. Specifically, we expect that: (H2a) environmental autocorrelation influences $\sum E_{a_{\mathrm{ij}}}^{\sigma^{2}}$ via its impact on population structure across timesteps. We base this prediction on the fact that the impact of environmental autocorrelation on population dynamics can be quantified by the degree to which the sequence of environments shifts the population from its long‐term mean stable state structure (Tuljapurkar and Haridas 2006). And lastly, we expect (H2b) environmental variance to influence $\sum E_{a_{\mathrm{ij}}}^{\sigma^{2}}$ via the populations' underlying demographic rates. This prediction also follows Tuljapurkar's approximation (1982, 1989), where the impact of environmental variance can be approximated by the summed products of demographic rate variances and sensitivity values.

## 2 Methods

First, we define the integral projection models (IPMs; Easterling, Ellner, and Dixon 2000) used in our analyses. Then, we outline the simulation‐based methodology along with how timeseries of environmental variability (variation in environmental variables) were used to generate the timeseries of IPM kernels exhibiting different levels of environmental stochasticity (variation in demographic rates). And finally, we detail how demographic buffering ($\sum E_{a_{\mathrm{ij}}}^{\sigma^{2}}$) was calculated along with the methods to infer its underlying demographic mechanisms.

### 2.1 Stochastic Integral Projection Models

To explore the drivers of demographic buffering, we used integral projection models (IPMs). IPMs are discrete time population models (i.e., they project populations across well‐defined intervals of time from t to t+1) that are structured with respect to a continuous variable (Easterling, Ellner, and Dixon 2000; Ellner, Childs, and Rees 2016). To investigate the environmental components and demographic mechanisms that impact degrees of demographic buffering in natural populations, we used environmentally explicit, parameter‐stochastic IPMs for the temperate deciduous shrub *Berberis thunbergii* (Merow et al. 2017) and the tropical herbaceous perennials *Calathea crotalifera* (Westerband and Horvitz 2017) and *Heliconia tortuosa* (Westerband and Horvitz 2017), extracted from the PADRINO IPM database (Levin et al. 2022). The chosen model structure allows us to individually influence regression parameters associated with environmental variables that underpin the IPM subkernels (i.e., the progression **P**‐ and fertility **F**‐subkernels) to test our hypotheses.

We chose these three published IPMs to compare the roles of environmental variables and $\lambda_{s}$ on $\sum E_{a_{\mathrm{ij}}}^{\sigma^{2}}$ to gain some generality. The *B. thunbergii* IPM uses five environmental variables to build its kernels: Mean temperature during the warmest month, mean May precipitation, photosynthetically active radiation (PAR), soil nitrogen and soil pH. The *C. crotalifera* and *H. tortuosa* IPMs use two environmental variables to define their kernels: Canopy openness and photosynthetic rate. The kernel structure, environmental variables and vital rate regressions for *B. thunbergii* , *C. crotalifera* and *H. tortuosa* are detailed in Tables S1–S3, respectively. Furthermore, the models inhabit different domains of $\lambda_{s}$. The models of *B. thunbergii* and *H. tortuosa* have values of $\lambda_{s}>1$ ( *B. thunbergii* : $\lambda_{s}=1.378$; *H. tortuosa* : $\lambda_{s}=1.367$), implying long‐term population growth, whilst *C. crotalifera* has $\lambda_{s}<1$ ($\lambda_{s}=0.976$), describing long‐term population decline (Figure S1). This combination of environmental variables and $\lambda_{s}$ values across the three species offers a unique opportunity for the exploration of demographic buffering in variable environments. Specifically, since *C. crotalifera* and *H. tortuosa* have the same environmental variables and *B. thunbergii* and *H. tortuosa* have highly similar $\lambda_{s}$ values, our study allows an exploration of demographic buffering whilst mitigating the potential confounds of environmental variables and $\lambda_{s}$.

### 2.2 Simulation Methodology

**FIGURE 1:**
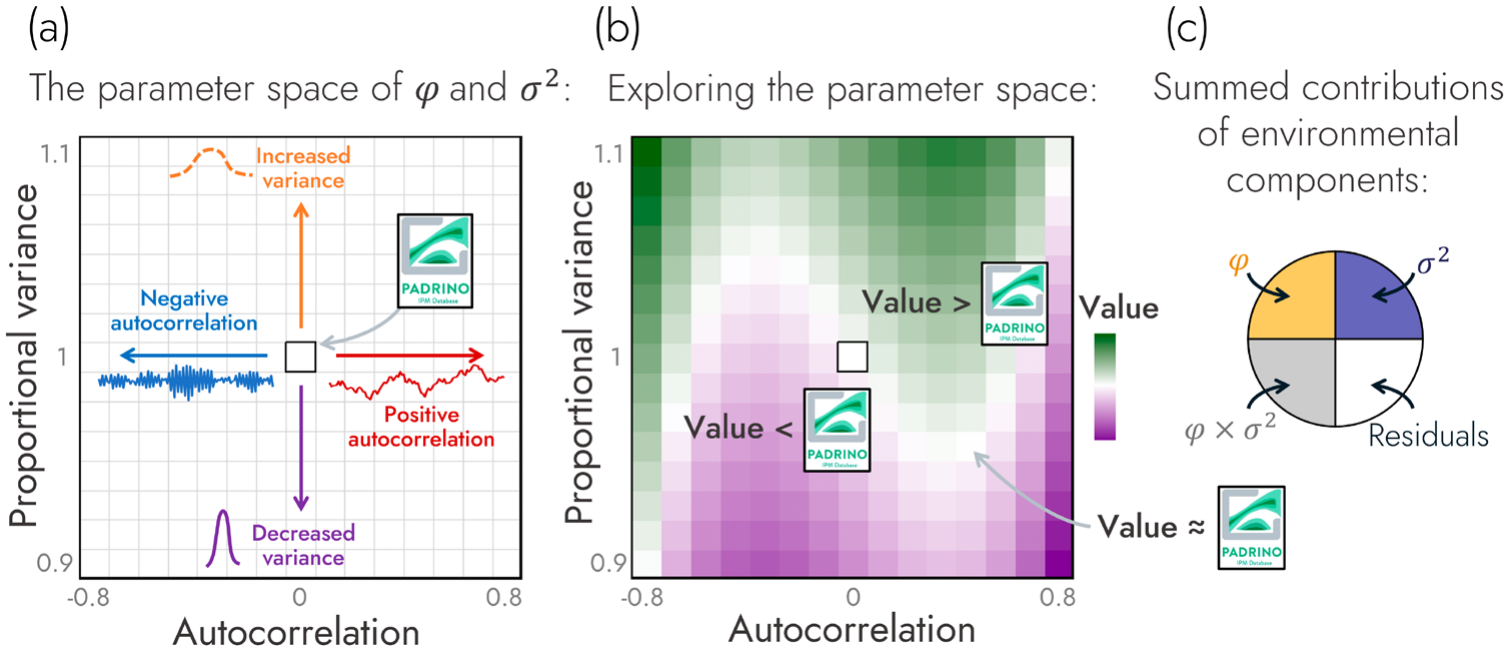
Overview of the simulation and analysis structure implemented to examine the impacts of environmental components on natural populations. In our simulations, we explored how a population's measure of demographic buffering changes over a parameter space of environmental autocorrelation and variance values. (a) This space is visualised here across a 2D surface with environmental autocorrelation on the *x*‐axis and proportional variance on the *y*‐axis. Environmental variance is noted as proportional variance which is defined as the relative increase (> 1) or decrease (< 1) in the variance of a climate driver made relative to the climate driver's variance value stored in the PADRINO database. The middle of this landscape (i.e., autocorrelation = 0 and proportional variance = 1) represents the population model stored in the PADRINO database. (b) Impacts of environmental autocorrelation and variance on a response variable (e.g., degree of demographic buffering or a measure of population structure) are shown projected as a third dimension across this landscape. Across this projection, values lower than those reported in the original PADRINO IPM model are coloured purple, values close to the PADRINO model are coloured white and values greater than the PADRINO model are coloured green. (c) The most parsimonious model that predicts the response variable as a function of environmental autocorrelation and proportional variance was retained to calculate the summed linear and curvilinear contributions of each predictor and the residuals towards the variance in the response variable.

To explore the roles of (H1) environmental components as well as (H2a) population structure and (H2b) demographic rates on demographic buffering, we simulated IPMs across an environmental autocorrelation—variance parameter space. In this simulation, all stochastic environmental parameters varied fully factorially across the axes of autocorrelation, from −0.8 to 0.8, and proportional variance, ranging from 0.9 (10% less variance) to 1.1 (10% more variance), relative to the IPM from PADRINO (Figure 1a,b). We used the species‐specific environmental variables to construct a timeseries of 1000 IPM kernels, for each species, from which we then estimated $\lambda_{s}$ (Equation 3). Specifically, to calculate $\lambda_{s}$: (1) a population of random structure was initialised, (2) the population was then projected through a series of 1000 IPM kernels and (3) population sizes from timesteps 200 to 1000 were used to calculate $\lambda_{s}$ following the equation:

(3) $$\lambda_{s}=\exp\left(E\left[\ln\left(\frac{N_{t+1}}{N_{t}}\right)\right]\right)$$

We omitted the first 200 projections from our calculation of $\lambda_{s}$ to mitigate the impacts of transient dynamics (McDonald et al. 2016).

### 2.3 Generating Environmental Timeseries

To explore how environmental components influence demographic buffering (H1), we manipulated the temporal autocorrelation and variance of environmental variables in our environmentally explicit, parameter‐stochastic IPMs. Whilst the effects of variance in demographic rates on population dynamics have been investigated in population ecology (Le Coeur et al. 2022; Drake 2005; Jackson, Le Coeur, and Jones 2022), the effects of temporal autocorrelation on population performance are much less explored, despite having broad impacts on population dynamics (Evers, Knight, and Compagnoni 2023; Petchey 2000; Petchey, Gonzalez, and Wilson 1997; Smallegange, Deere, and Coulson 2014), life histories (Paniw, Ozgul, and Salguero‐Gómez 2018; Vinton et al. 2023) and evolution (Vinton et al. 2022; Wieczynski, Turner, and Vasseur 2018). To fill this gap in knowledge, we used a first‐order autoregressive function to generate the sequence of environment values used to build multiple timeseries of IPM kernels exhibiting different levels of autocorrelation in demographic rates. Here, φ represents the degree of autocorrelation across timesteps, whilst $\epsilon_{t+1}$ represents white noise (i.e., random draws from a normal distribution, $\epsilon \sim N\left(0,1\right)$).

(4) $$X_{t+1}=\varphi X_{t}+\epsilon_{t+1}$$

Subsequently, the sequence of autocorrelated values was shifted and scaled to a desired mean $\left(\mu \right)$ and variance $\left(\sigma^{2}\right)$ to generate the simulated environmental timeseries (see Tables S1–S3):

(5) $$\text{Environment}=\left[\frac{\sqrt{\sigma^{2}}\left[X-\text{mean}\left(X\right)\right]}{\sqrt{\mathrm{var}\left(X\right)}}\right]+\mu$$

As our objective is not to evaluate the effect of shifts in mean environment values on demographic buffering, μ values were kept constant across simulations, whilst $\sigma^{2}$ values varied across simulations.

Since the environmental variables across the three species have different variances, we standardised the increase/decrease in environmental variance across environmental variables. Specifically, we manipulated variances proportionally $\left(\sigma_{\text{prop}.}^{2}\right)$ with respect to their variances coded in the PADRINO database $\left(\sigma_{\text{init}.}^{2}\right)$ (Levin et al. 2022).

(6) $$\sigma^{2}=\sigma_{\text{init}.}^{2}\sigma_{\text{prop}.}^{2}$$

This proportional increase/decrease in environmental variance subsequently generates a timeseries of IPM kernels exhibiting different levels of variance in demographic rates.

Generating these environmental timeseries creates a parameter space of IPM kernel environmental stochasticity with axes of temporal autocorrelation and proportional variance in demographic rates. It is worth noting that this parameter space does not represent a realised scenario. Instead, the purpose of this parameter space is to manipulate the degrees of environmental variability across two axes to inform how demographic buffering responds across these axes (Figure 1b).

### 2.4 Analysing the Effects of Environmental Autocorrelation and Variance

To explore the effects of environmental components on demographic buffering (H1 and H2), we constructed a suite of linear models using autocorrelation and proportional variance as predictors. Since the impacts of autocorrelation and proportional variance on demographic buffering may be curvilinear, we constructed models using the linear, quadratic and cubic forms of proportional variance and autocorrelation as predictors. Furthermore, since the linear effects of autocorrelation and proportional variance on $\sum E_{a_{\mathrm{ij}}}^{\sigma^{2}}$ may depend on one another (Roughgarden 1975; Vinton et al. 2022), we included the product of both environmental components (i.e., autocorrelation × proportional variance) as an interaction term in our model selection. To select the most parsimonious model, we used model comparison based on AIC (see Supporting Information p. 10 for the full analysis pipeline and Tables S4–S12 for the full AIC breakdown). From the selected model, we calculated the proportion of variance in $\sum E_{a_{\mathrm{ij}}}^{\sigma^{2}}$ that can be explained by the full model (*R* ^2^) along with the summed contributions of autocorrelation, proportional variance, autocorrelation × proportional variance and residuals (Figure 1c). These contributions were calculated by taking the sums of squares associated with each predictor and dividing them by the total sum of squares associated with the selected model.

### 2.5 Perturbation Analyses to Quantify $\sum E_{a_{\mathrm{ij}}}^{\sigma^{2}}$

To quantify the degree of demographic buffering across our simulations (testing H1 and H2), we calculated the summation of stochastic elasticities of variance of $\lambda_{s}$ with respect to demographic rates. We estimated this variable, $\sum E_{a_{\mathrm{ij}}}^{\sigma^{2}}$, *numerically*. Whilst the **K**‐kernel of an IPM is defined as a continuous density function that projects a continuously structured population across discrete timesteps, in practice, we discretise the kernel into a matrix notated as A (Easterling, Ellner, and Dixon 2000; Ellner, Childs, and Rees 2016). Since A is composed of the individual matrix elements $a_{\mathrm{ij}}$ which represent individual demographic rates (i.e., both the survival‐dependent transitions of individuals from stage j to stage i and the per‐capita reproductive contributions of individuals in stage j to stage i across timesteps) and our stochastic environment generates a temporal sequence of A matrices ($A_{t}$), we can quantify the temporal variance of each $a_{\mathrm{ij}}$ element across $A_{t}$—i.e., $\mathrm{var}\left(a_{\mathrm{ij},t}\right)$. In turn, we numerically calculated $\sum E_{a_{\mathrm{ij}}}^{\sigma^{2}}$ by perturbing the temporal variance of each matrix element's timeseries $\left(a_{\mathrm{ij},t}\right)$ by 0.00001, proportionate to the unperturbed temporal variance of $a_{\mathrm{ij},t}$. After the perturbation, we calculated a perturbed stochastic population growth rate associated with the perturbed element's timeseries $\left(\lambda_{s}^{*a_{\mathrm{ij},t}}\right)$. The summation of these weighted differences in $\lambda_{s}$ and $\lambda_{s}^{*a_{\mathrm{ij},t}}$ yields $\sum E_{a_{\mathrm{ij}}}^{\sigma^{2}}$.

(7) $$\sum E_{a_{\mathrm{ij}}}^{\sigma^{2}}=\sum \left[\frac{\mathrm{var}\left(a_{\mathrm{ij},t}\right)}{\lambda_{s}}\times \frac{\lambda_{s}^{*a_{\mathrm{ij},t}}-\lambda_{s}}{0.00001\times \mathrm{var}\left(a_{\mathrm{ij},t}\right)}\right]$$

To calculate the impact of demographic rates on demographic buffering (H2b), we perturbed the subkernels that describe survival‐dependent changes in size (**P**), termed progression herein, and fertility (**F**) using the same method we used for the **K**‐kernels. After calculating the subkernel‐level elasticities of variance (Griffith 2017), we subtracted the subkernel summed elasticities of variance to calculate their relative contributions: **P–F** contribution. Positive (negative) values of **P–F** contribution indicate that proportional increases in the variance of progression rates are more (less) impactful on $\lambda_{s}$ than proportional increases in the variance of fertility rates.

### 2.6 Quantifying the Impact of Population Structure on $\sum E_{a_{\mathrm{ij}}}^{\sigma^{2}}$

To analyse how population structure influences demographic buffering (H2a), we used two numerical approaches. Whilst methods exist to *analytically* measure the impact of population structure on the asymptotic properties of population dynamics (Tuljapurkar and Lee 1997), currently there are no analytical approaches to quantify the degree to which multiple environmental components influence $\sum E_{a_{\mathrm{ij}}}^{\sigma^{2}}$ via population structure. In turn, we developed two approaches: A *deviance‐based approach* and a *size‐based approach*. These approaches *numerically* link the impacts of environmental autocorrelation and variance on $\sum E_{a_{\mathrm{ij}}}^{\sigma^{2}}$ to population structure. Importantly, using these two approaches to investigate H2a allows us to cross‐validate outputs—i.e., the hypothesised result of environmental autocorrelation impacting $\sum E_{a_{\mathrm{ij}}}^{\sigma^{2}}$ via shifts in population structure.

The *deviance‐based approach* involved examining deviances from stationary distributions. To do so, we first quantified the expected buffering value ($\sum E_{a_{\mathrm{ij}}}^{\sigma^{2}}|\mathrm{ASD}$) of individuals in the population. This expected buffering value is the average of the buffering value associated with each stage ($\sum_{j}E_{a_{\mathrm{ij}}}^{\sigma^{2}}$) weighted by the proportion of individuals in that stage, relative to the population's average size distribution (ASD). To determine the population's average size distribution for a given environment, we iterated 1000 randomly generated size distributions through the series of stochastic kernels and retained the mean of all size distributions across timesteps 200 to 1000. Burning‐in the first 200 timesteps mitigates the impact of transients on the ASD (McDonald et al. 2016). After calculating the values of $\sum E_{a_{\mathrm{ij}}}^{\sigma^{2}}|\mathrm{ASD}$ across the parameter space of environmental autocorrelation and variance, we quantified the degree to which variance in expected buffering values deviated from the variances in $\sum E_{a_{\mathrm{ij}}}^{\sigma^{2}}$—i.e., $\sum E_{a_{\mathrm{ij}}}^{\sigma^{2}}|\mathrm{ASD}-\sum E_{a_{\mathrm{ij}}}^{\sigma^{2}}$. To quantify this deviation, we calculated the difference between $\sum E_{a_{\mathrm{ij}}}^{\sigma^{2}}|\mathrm{ASD}$ and $\sum E_{a_{\mathrm{ij}}}^{\sigma^{2}}$ where both $\sum E_{a_{\mathrm{ij}}}^{\sigma^{2}}|\mathrm{ASD}$ and $\sum E_{a_{\mathrm{ij}}}^{\sigma^{2}}$ were scaled (mean = 0, standard deviation = 1). Deviances of $\sum E_{a_{\mathrm{ij}}}^{\sigma^{2}}|\mathrm{ASD}-\sum E_{a_{\mathrm{ij}}}^{\sigma^{2}}$ from 0 (i.e., scenarios where $\sum E_{a_{\mathrm{ij}}}^{\sigma^{2}}|\mathrm{ASD}\neq \sum E_{a_{\mathrm{ij}}}^{\sigma^{2}}$ across the environmental autocorrelation—variance parameter space) indicate that shifts in population structure may influence $\sum E_{a_{\mathrm{ij}}}^{\sigma^{2}}$. Subsequently, regressing these deviances against the environmental components allows us to implicate an environmental component, hypothesised to be environmental autocorrelation (H2a), as driving the impact of population structure on $\sum E_{a_{\mathrm{ij}}}^{\sigma^{2}}$.

The *size‐based approach* involved calculating the mean of the distribution of demographic buffering across a life history, termed *mean buffered size*. Calculating mean buffered size allows us to explore if the degree of buffering across a life history is shifted towards smaller or larger sizes across the environmental autocorrelation—variance parameter space. To calculate this mean buffered size, we calculated the relative size (i.e., 0 = smallest possible size (*α*) and 1 = maximum possible size (*ω*)) that corresponds to the centre of the distribution of $\sum E_{a_{\mathrm{ij}}}^{\sigma^{2}}$ across the domain of sizes (Equation 8). This calculation mirrors the method of calculating generation time as the mean age of reproductive individuals in a population (Ebert 1999, 14).

(8) $$\text{Mean buffered size}=\frac{1}{\omega }\left[\frac{\sum_{j}\left(j\sum_{i}E_{a_{\mathrm{ij}}}^{\sigma^{2}}\right)\;}{\sum E_{a_{\mathrm{ij}}}^{\sigma^{2}}\;}-\alpha \right]$$

After calculating the mean buffered size for each simulated population across the environmental autocorrelation—variance parameter space, we regressed mean buffered size against the environmental components to test our hypothesis that environmental autocorrelation influences $\sum E_{a_{\mathrm{ij}}}^{\sigma^{2}}$ via shifts in population structure (H2a).

## 3 Results

### 3.1 Testing H1: Environmental Variance Is the Primary Driver of Demographic Buffering

**FIGURE 2:**
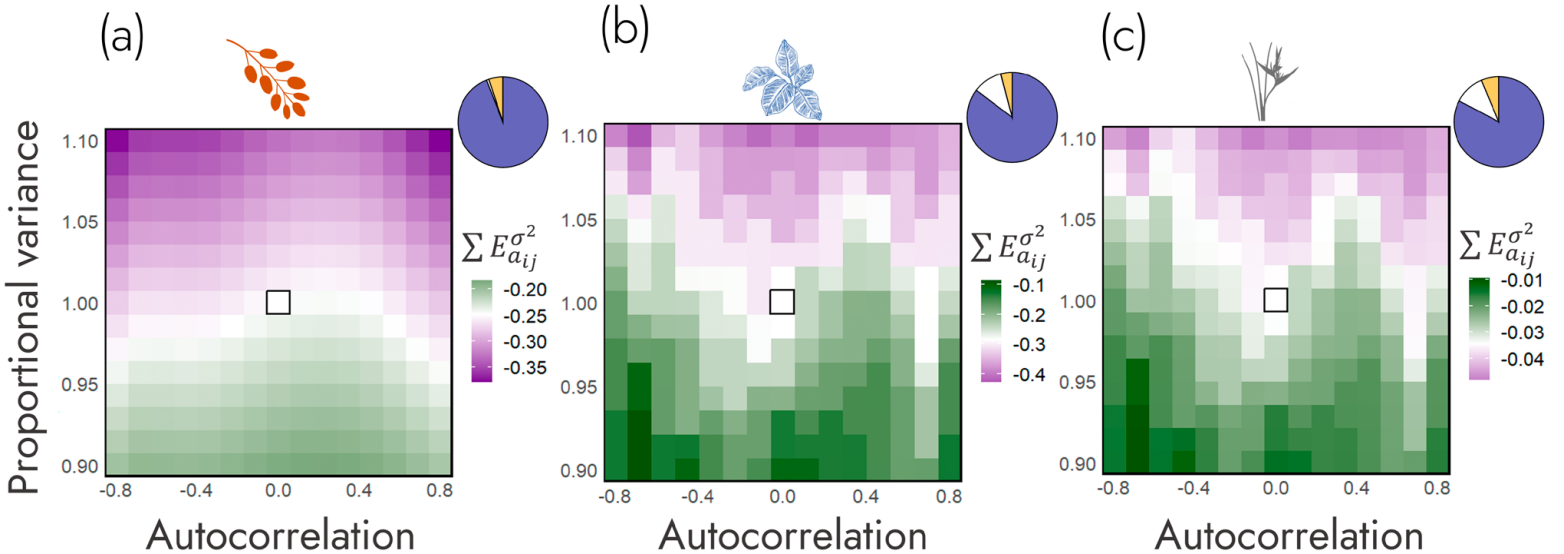
Environmental variance is the primary driver of demographic buffering. Across *Berberis thunbergii* (a), *Calathea crotalifera* (b) and *Heliconia tortuosa* (c), environmental variance (blue in pie‐chart) explains the majority of variance in $\sum E_{a_{\mathrm{ij}}}^{\sigma^{2}}$. Populations of all three species become relatively less buffered (lower values of $\sum E_{a_{\mathrm{ij}}}^{\sigma^{2}}$, in purple) as proportional variance in the environment increases, whilst populations become relatively more buffered (higher values of $\sum E_{a_{\mathrm{ij}}}^{\sigma^{2}}$, in green) as environmental variance decreases. This strong impact of proportional variance is summarised in the pie charts detailing the proportion of variance in $\sum E_{a_{\mathrm{ij}}}^{\sigma^{2}}$ that can be explained by the environmental components: Environmental autocorrelation in orange, environmental variance in blue, environmental autocorrelation × variance interaction in grey (so small here that it is not visible) and unexplained residuals in white. Since the pie charts are predominantly blue across all three species, proportional variance is the primary driver of $\sum E_{a_{\mathrm{ij}}}^{\sigma^{2}}$ across the environmental autocorrelation—variance parameter space.

Here, we tested the hypothesis that environmental autocorrelation and variance have negative effects on demographic buffering as quantified via $\sum E_{a_{\mathrm{ij}}}^{\sigma^{2}}$ (H1). To do so, we ran simulations of the *Berberis thunbergii* , *Calathea crotalifera* and *Heliconia tortuosa* IPMs across a domain of autocorrelation and proportional variance values and calculated $\sum E_{a_{\mathrm{ij}}}^{\sigma^{2}}$. We found environmental variance to be the primary driver of variance in $\sum E_{a_{\mathrm{ij}}}^{\sigma^{2}}$ (Figure 2). The summed contributions of proportional variance accounted for 94% of the variance of $\sum E_{a_{\mathrm{ij}}}^{\sigma^{2}}$ in *B. thunbergii* (*R* ^2^ = 0.99, Figure 2a, Table S4), 85% of the variance of $\sum E_{a_{\mathrm{ij}}}^{\sigma^{2}}$ in *C. crotalifera* (*R* ^2^ = 0.89, Figure 2b, Table S5) and 83% of the variance of $\sum E_{a_{\mathrm{ij}}}^{\sigma^{2}}$ in *H. tortuosa* (*R* ^2^ = 0.89, Figure 2c, Table S6). Supporting our hypothesis, environmental variance had a negative effect on $\sum E_{a_{\mathrm{ij}}}^{\sigma^{2}}$ (see models for *B. thunbergii* , *C. crotalifera* and *H. tortuosa* in Tables S4–S6). However, we did not find evidence for a simple negative effect of environmental autocorrelation on $\sum E_{a_{\mathrm{ij}}}^{\sigma^{2}}$. All species were best modelled when the linear, quadratic and cubic forms of autocorrelation were used as predictors of $\sum E_{a_{\mathrm{ij}}}^{\sigma^{2}}$. These findings indicate the impact of autocorrelation on $\sum E_{a_{\mathrm{ij}}}^{\sigma^{2}}$ is curvilinear across the environmental autocorrelation—variance parameter space.

### 3.2 Testing H2a: Temporal Autocorrelation Influences Demographic Buffering via Population Structure

We used two approaches to test the hypothesis that temporal autocorrelation influences demographic buffering via shifts in population structure (H2a). First, we used a *deviance‐based approach* which used the differences between demographic buffering weighted by population structure ($\sum E_{a_{\mathrm{ij}}}^{\sigma^{2}}|\mathrm{ASD}$) and $\sum E_{a_{\mathrm{ij}}}^{\sigma^{2}}$. Second, we used a *size‐based approach* which quantified shifts in the distribution of buffering across the life history.

**FIGURE 3:**
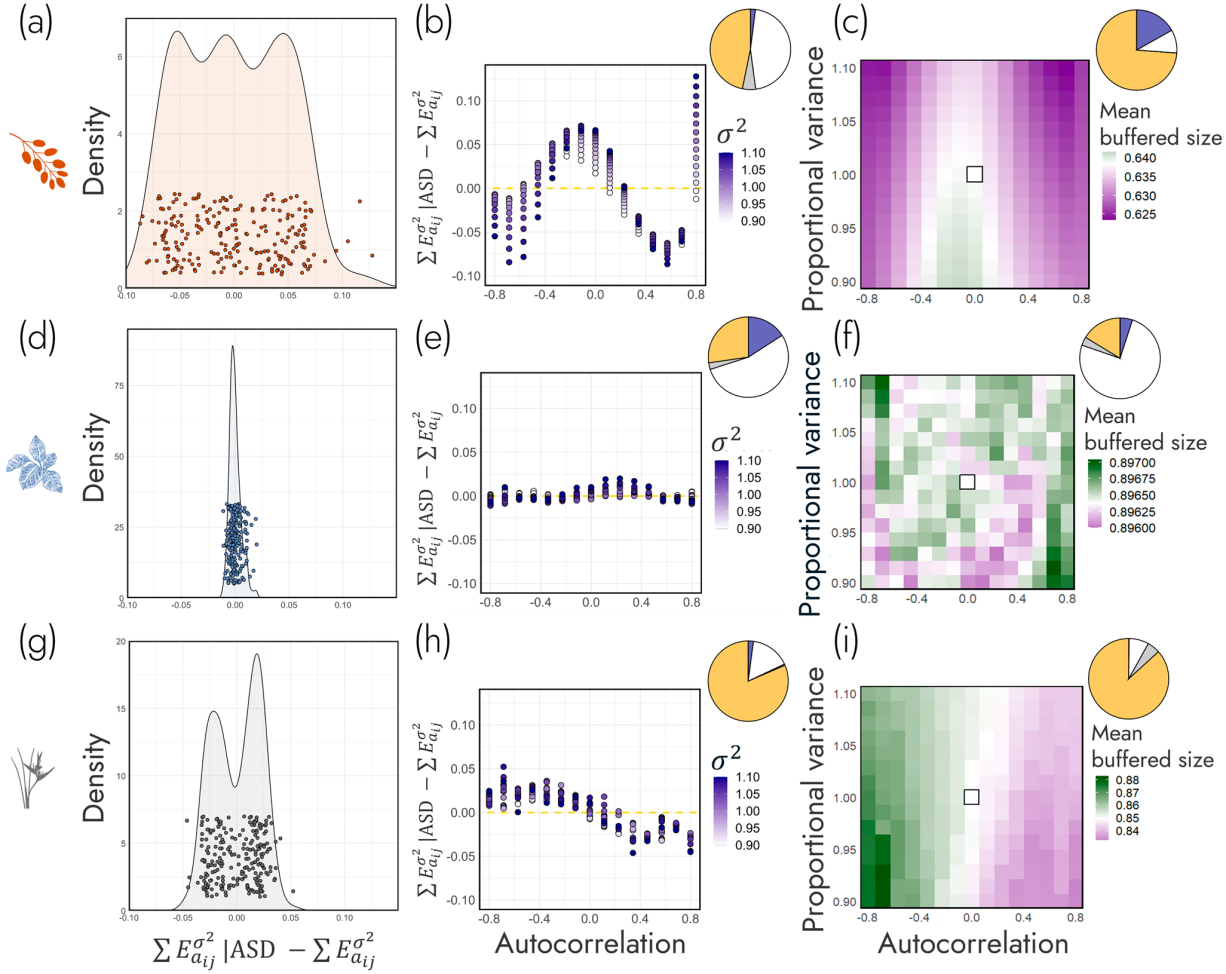
Environmental autocorrelation can influence demographic buffering ($\sum E_{a_{\mathrm{ij}}}^{\sigma^{2}}$) via its impact on population structure. In addition, the degree to which environmental autocorrelation impacts $\sum E_{a_{\mathrm{ij}}}^{\sigma^{2}}$ across *Berberis thunbergii* (a–c), *Calathea crotalifera* (d–f) and *Heliconia tortuosa* (g–i) is species‐specific. The first column (a, d, g) shows the distribution of differences between $\sum E_{a_{\mathrm{ij}}}^{\sigma^{2}}$ and demographic buffering weighted by the average stage distribution ($\sum E_{a_{\mathrm{ij}}}^{\sigma^{2}}|\mathrm{ASD}$) across the environmental autocorrelation—variance parameter space. These differences from 0 show the potential impact of population structure on $\sum E_{a_{\mathrm{ij}}}^{\sigma^{2}}$. We then, in the second column (b, e, h), investigate these differences as a function of environmental autocorrelation (*x*‐axis) and environmental variance ($\sigma^{2}$, purple). Lastly, in the third column (c, f, i), we quantify the impact of environmental autocorrelation and variance on the mean buffered size of the population. The pie charts at the top right‐hand corner of panels in (b, e, h) and (c, f, i) detail the proportion of variance in the response variable that is explained by environmental autocorrelation (orange), environmental variance (blue), environmental autocorrelation × variance interaction (grey) and residuals (white). These pie charts show how environmental autocorrelation primarily drives shifts in $\sum E_{a_{\mathrm{ij}}}^{\sigma^{2}}$ through population structure.

In our *deviance‐based approach*, we subtracted scaled values of $\sum E_{a_{\mathrm{ij}}}^{\sigma^{2}}$ across all simulations against their respective $\sum E_{a_{\mathrm{ij}}}^{\sigma^{2}}$ normalised by simulation specific average size distributions ($\sum E_{a_{\mathrm{ij}}}^{\sigma^{2}}|\mathrm{ASD}$). Since both values are scaled to mean = 0 with standard deviation = 1, any deviation of $\sum E_{a_{\mathrm{ij}}}^{\sigma^{2}}|\mathrm{ASD}-\sum E_{a_{\mathrm{ij}}}^{\sigma^{2}}$ from 0 indicates that temporal shifts in population structure may impact demographic buffering. Interestingly, we found heterogeneity in the degree to which $\sum E_{a_{\mathrm{ij}}}^{\sigma^{2}}|\mathrm{ASD}$ differed from $\sum E_{a_{\mathrm{ij}}}^{\sigma^{2}}$ across species. Specifically, we found a hierarchy of variance in $\sum E_{a_{\mathrm{ij}}}^{\sigma^{2}}|\mathrm{ASD}-\sum E_{a_{\mathrm{ij}}}^{\sigma^{2}}$ values where *B. thunbergii* has the most variance (SD = 0.0477, Figure 3a), *H. tortuosa* has moderate variance (SD = 0.0215, Figure 3g) and *C. crotalifera* has the least variance (SD = 0.00536, Figure 3d). This heterogeneity suggests that population structure may influence $\sum E_{a_{\mathrm{ij}}}^{\sigma^{2}}$ in *B. thunbergii* and *H. tortuosa* to a greater degree than *C. crotalifera* .

To determine if environmental autocorrelation is driving these variances, we modelled $\sum E_{a_{\mathrm{ij}}}^{\sigma^{2}}|\mathrm{ASD}-\sum E_{a_{\mathrm{ij}}}^{\sigma^{2}}$ values in response to environmental autocorrelation and variance. Supporting our hypothesis (H2a), we found the variance in $\sum E_{a_{\mathrm{ij}}}^{\sigma^{2}}|\mathrm{ASD}-\sum E_{a_{\mathrm{ij}}}^{\sigma^{2}}$ values is mostly explained by environmental autocorrelation (Figure 3b,e,h). In *B. thunbergii* and *H. tortuosa* , environmental autocorrelation accounted for 48% (*R* ^2^ = 0.56, Figure 3b, Table S7) and 81% (*R* ^2^ = 0.84, Figure 3h, Table S9) of the variance in $\sum E_{a_{\mathrm{ij}}}^{\sigma^{2}}|\mathrm{ASD}-\sum E_{a_{\mathrm{ij}}}^{\sigma^{2}}$ values, respectively; whilst environmental variance only accounted for 2% of the variance in both species. Regarding *C. crotalifera* , the largest contributor to variance in $\sum E_{a_{\mathrm{ij}}}^{\sigma^{2}}|\mathrm{ASD}-\sum E_{a_{\mathrm{ij}}}^{\sigma^{2}}$ values was unexplained residual variance (56%, *R* ^2^ = 0.47, Figure 3e, Table S8), followed by environmental autocorrelation (28%) and variance (16%).

In our *size‐based approach*, we analysed the impact of environmental autocorrelation and variance on the distribution of demographic buffering across a life cycle. In turn, we calculated the centre of the distribution of demographic buffering across a life history: Mean buffered size. Echoing the findings from the first line of enquiry, mean buffered size was best explained by changes in environmental autocorrelation—especially in *B. thunbergii* and *H. tortuosa* . Specifically, 74% and 91% of the variance in mean buffered size was attributed to environmental autocorrelation in *B. thunbergii* (*R* ^2^ = 0.91, Figure 3c, Table S10) and *H. tortuosa* (*R* ^2^ = 0.97, Figure 3i, Table S12), respectively; whilst environmental variance only accounted for 17% and 0.1% of the variance in mean buffered size, respectively. However, just as in the first line of enquiry, $\sum E_{a_{\mathrm{ij}}}^{\sigma^{2}}$ in *C. crotalifera* is less exposed to impacts of shifts in population structure as the distribution of mean buffered size across the environmental autocorrelation—variance parameter space was mostly explained by residual variance (78%) rather than environmental autocorrelation (17%) or environmental variance (5%) (*R* ^2^ = 0.26, Figure 3f, Table S11).

### 3.3 Testing H2b: Demographic Buffering Is Most Sensitive to Environmental Variance's Impact on Rates of Progression and Fertility

To test the hypothesis that environmental variance impacts demographic buffering through vital rates (H2b), we ran the same perturbation analysis used to calculate $\sum E_{a_{\mathrm{ij}}}^{\sigma^{2}}$ at the level of the subkernels: **P**‐subkernel (progression) and the **F**‐subkernel (fertility). By taking the difference of the subkernel elasticities of variance (i.e., **P–F** contribution), we investigated (1) the role of underlying rates of progression and fertility on demographic buffering and (2) the environmental components that influence the **P–F** contribution across the environmental autocorrelation—variance parameter space.

**FIGURE 4:**
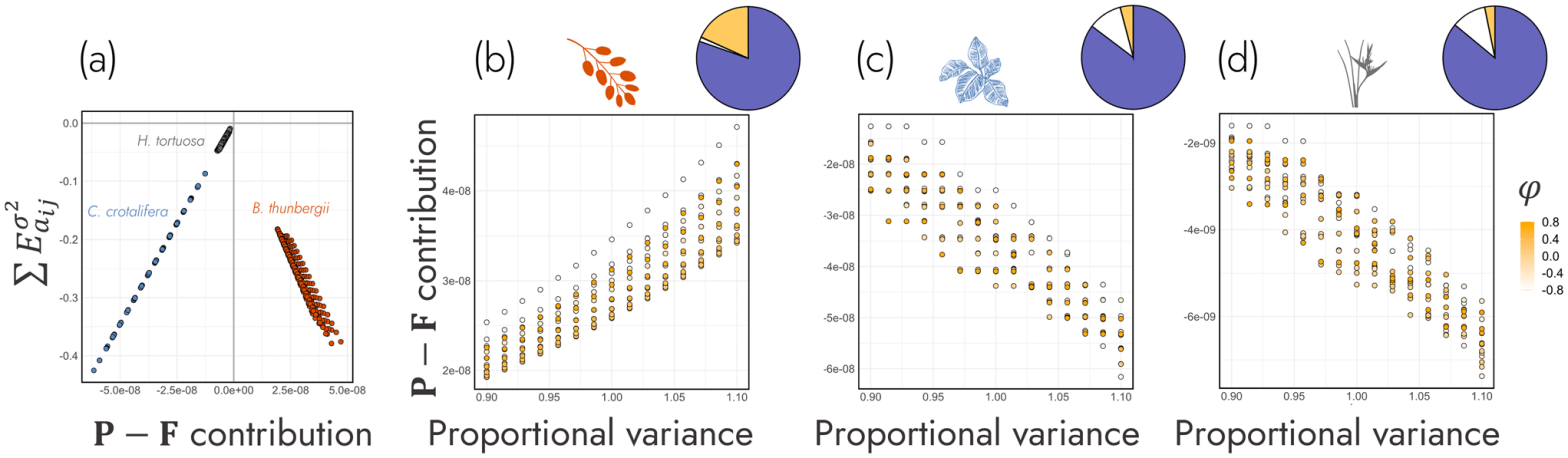
Environmental variance influences demographic buffering ($\sum E_{a_{\mathrm{ij}}}^{\sigma^{2}}$) via the population's underlying demographic rates. (a) The relative contribution of progression (growth conditional on survival: **P**) and fertility (recruitment of new individuals from reproductive ones the previous timestep: **F**) on $\sum E_{a_{\mathrm{ij}}}^{\sigma^{2}}$ (i.e., **P**–**F** contribution) was calculated for three plant species: (b) *Berberis thunbergii* , (c) *Calathea crotalifera* and (d) *Heliconia tortuosa*. Dots are coloured by the degree of environmental autocorrelation (φ, yellow). The pie charts at the top right‐hand corner of panels (b)–(d) detail the proportion of variance in **P**–**F** contribution that is explained by environmental autocorrelation (orange), environmental variance (blue), environmental autocorrelation × variance interaction (grey) and residuals (white). These pie charts show how environmental variance is the primary driver of shifts in the relative contributions of progression and fertility to $\sum E_{a_{\mathrm{ij}}}^{\sigma^{2}}$.

First, we determined if **P–F** contribution is a sufficient predictor of $\sum E_{a_{\mathrm{ij}}}^{\sigma^{2}}$. **P–F** contribution was highly predictive of $\sum E_{a_{\mathrm{ij}}}^{\sigma^{2}}$ across all species (Figure 4a). *B. thunbergii* had a negative relationship between **P–F** contribution and $\sum E_{a_{\mathrm{ij}}}^{\sigma^{2}}$ (*r*(223) = −0.968, *p* < 0.001), whilst *C. crotalifera* and *H. tortuosa* had positive relationships ( *C. crotalifera* : *r*(223) = 0.999, *p* < 0.001; *H. tortuosa* : *r*(223) = 0.983, *p* < 0.001). These results indicate lower degrees of demographic buffering are associated with a greater impact of variance in rates of progression (vs. fertility) in *B. thunbergii* but the opposite, a greater impact of variance in fertility (vs. progression), in *C. crotalifera* and *H. tortuosa* .

To test if variance in **P–F** contribution is most explained by environmental variance rather than autocorrelation (H2b), we regressed **P–F** contribution against the environmental components. Across the three species, **P–F** contribution was mostly explained by environmental variance rather than environmental autocorrelation (Figure 4b–d). Specifically, environmental variance explained 80%, 85% and 86% of the variance of **P–F** contribution in *B. thunbergii* (*R* ^2^ = 0.99, Figure 4b, Table S13), *C. crotalifera* (*R* ^2^ = 0.89, Figure 4c, Table S14) and *H. tortuosa* (*R* ^2^ = 0.89, Figure 4d, Table S15), respectively. However, environmental autocorrelation explained 18%, 4% and 3% of the variance of **P–F** contribution, respectively.

## 4 Discussion

Environmental drivers and demographic mechanisms are key to quantify and predict a population's capacity for demographic buffering. Using three stochastic IPMs from the PADRINO database (Levin et al. 2022), we obtain partial support for the hypothesis that environmental autocorrelation and variance negatively impact a population's capacity to remain demographically buffered (H1). Interestingly, whilst environmental variance negatively affects demographic buffering, there is a curvilinear effect of temporal autocorrelation on demographic buffering. Furthermore, we show that environmental autocorrelation and variance impact demographic buffering through different mechanisms. Temporal autocorrelation impacts demographic buffering ($\sum E_{a_{\mathrm{ij}}}^{\sigma^{2}}$) through shifts in population structure (H2a), whilst environmental variance impacts $\sum E_{a_{\mathrm{ij}}}^{\sigma^{2}}$ via underlying demographic rates (H2b). Specifically, the influence of environmental variance on rates of progression vs. fertility is the greatest driver of differences in $\sum E_{a_{\mathrm{ij}}}^{\sigma^{2}}$ across variable environments in the three examined species. This finding builds on multiple lines of evidence showing how different life histories can persist in variable environments via the differential variance of progression vs. fertility rates (Gaillard, Festa‐Bianchet, and Yoccoz 1998; Pfister 1998).

Identifying the mechanisms that underpin the ability of natural populations to buffer against environmental stochasticity offers a powerful framework to explore a population's vulnerability to climate change. Current climatic forecasts predict changes in environmental variability due to global climate change (Lewis and King 2017; Masson‐Delmotte et al. 2021; Shen et al. 2011). For example, periods of extreme variation in temperature and precipitation are expected to increase in the tropics and sub‐tropics which host the highest biodiversity (temperature: Bathiany et al. 2018; precipitation: Trenberth 2011). Furthermore, extreme weather events are expected to become more common, leading to increased autocorrelation (e.g., tropical cyclones: Knutson et al. 2010; fire frequency: Halofsky, Peterson, and Harvey 2020). However, environmental components impact populations in different ways (Hoffmann and Bridle 2022; Vinton et al. 2022, 2023). Part of this heterogeneity arises due to the shape of demographic rates across a life history varying widely across the tree of life (Healy et al. 2019; Jones et al. 2014; Paniw, Ozgul, and Salguero‐Gómez 2018; Salguero‐Gómez, Jones, Jongejans, et al. 2016; Varas‐Enriquez, van Daalen, and Caswell 2022). Further heterogeneity arises via the variety of environment‐demographic rate reaction norms expressed across taxa (Murren et al. 2014). Our framework provides a promising avenue to incorporate this heterogeneity for informed analyses of how environmental stochasticity impacts a population's demographic buffering capacity (Gascoigne, Kajin, and Salguero‐Gómez 2023). Whilst this study has primarily focused on environmental stochasticity rather than the impacts of curvilinear environment‐demographic rate reaction norms, our framework can be readily scaled for the inclusion of reaction norms (see Le Coeur et al. 2022).

Our results highlight an interesting, but often overlooked, role of population structure in demographic buffering. Whilst we find environmental autocorrelation to primarily impact demographic buffering via shifts in population structure, there is also species‐level heterogeneity in the strength and direction by which environmental autocorrelation shifts population structure. One likely source of this heterogeneity is transient dynamics (i.e., short‐term, progressively weakening realisations of non‐asymptotic λ values resulting from a population not being at its stable‐stage distribution (Stott, Townley, and Hodgson 2011)). Whilst transient dynamics represent a suite of different stereotyped population dynamics (Capdevila et al. 2020, 2022), only *reactivity* (the degree to which a population not at its stable‐stage distribution increases/decreases relative to that same population projected from its stable‐stage distribution (Neubert and Caswell 1997)) has been linked to stochastic demography (McDonald et al. 2016; but see Tuljapurkar et al. 2023). However, the link between reactivity, along with other transient dynamics, and demographic buffering remains unknown. Future work analysing how transient dynamics influence levels of demographic buffering will finally integrate the analysis of transient dynamics with stochastic demography.

In addition to transient dynamics, future studies should examine the effects of demographic stochasticity as a potential mechanism underlying demographic buffering. Demographic stochasticity is defined as the variance in population‐level outcomes arising due to probabilistic realisations of demographic rates within individuals (Engen, Bakke, and Islam 1998; Melbourne and Hastings 2008). Importantly, the contribution of demographic stochasticity to long‐term population dynamics increases as population size decreases (Engen, Bakke, and Islam 1998). However, the opposite is true for environmental stochasticity. The role of environmental stochasticity, relative to demographic stochasticity, increases as population size increases (Engen, Bakke, and Islam 1998). Therefore, whilst our framework demonstrates how environmental autocorrelation and variance impact demographic buffering through population structure and demographic rates, respectively, these relationships likely weaken and possibly change as the population size declines. Consequently, linking demographic buffering to demographic stochasticity remains a timely avenue of future research. We argue that this approach will be particularly important for endangered populations where the population size is, by definition, small.

Our framework offers a valuable tool for the study of life histories in stochastic environments. Historically, studies of life histories in stochastic environments have followed two branches: Modelling and dimension reduction. Modelling life histories in stochastic environments, whereby analytic or numeric methods are used for demographic inference in individual populations, has progressively put to rest some key problems within life history theory (iteroparity: Orzack and Tuljapurkar 1989, Tuljapurkar, Gaillard, and Coulson 2009; diapause: Tuljapurkar and Istock 1993; migration: Wiener and Tuljapurkar 1994; biennialism: Klinkhammer and de Jong 1983, Roerdink 1988, 1989; homeostasis: Orzack 1985; lability: Koons et al. 2009, Jongejans et al. 2010, Barraquand and Yoccoz 2013). However, one of the limitations of a modelling approach is losing the realism captured within the constraints, phylogenetic history and selection gradients that drive variance patterns in demographic rates.

From the empirical side, researchers have used dimension reduction techniques to unmask the patterns life histories exhibit in variable environments. Dimension reduction techniques, such as phylogenetically controlled principal component analyses (Revell 2012), are especially useful as a life history is not a value nor an object; a life history strategy is an abstract concept that researchers probe with life history traits—such as longevity, age at sexual maturity, average body size, etc. To capture the signal of an individual life history strategy through the dimensionality, reducing the multidimensionality of life history metrics to their most important axes of variance (i.e., principal components) has led to key discoveries (two axes of life history variance (Salguero‐Gómez, Jones, Jongejans, et al. 2016; Healy et al. 2019)). Furthermore, this approach has been used to model life histories in stochastic environments (Paniw, Ozgul, and Salguero‐Gómez 2018; Rademaker, van Leeuwen, and Smallegange 2024; Romeijn and Smallegange 2022). However, this approach has been limited to modelling only one component of a variable environment (e.g., environmental autocorrelation *or* variance). This limitation is further emphasised by our results showing curvilinear relationships in the effects of environmental components on $\sum E_{a_{\mathrm{ij}}}^{\sigma^{2}}$, thereby illustrating that the impact of an environmental component on a demographic process is context‐dependent.

Using our framework, researchers can stitch the modelling and dimension reduction approaches together. Our framework can be applied to any environmentally explicit structured population model: from physiologically structured population models (de Roos 1997) to matrix population models (Caswell 2001) to integral projection models (Easterling, Ellner, and Dixon 2000; Ellner, Childs, and Rees 2016), to dynamic energy budget models (Nisbet et al. 2000; Smallegange et al. 2017). By using open‐access data (COMPADRE: Salguero‐Gómez et al. 2015; COMADRE: Salguero‐Gómez, Jones, Archer, et al. 2016; PADRINO: Levin et al. 2022; AmP: Marques et al. 2018), researchers can explore the combined impact of autocorrelation and variance on $\sum E_{a_{\mathrm{ij}}}^{\sigma^{2}}$ by interfacing the timeseries of a structured population model with stochastic matrices (Paniw, Ozgul, and Salguero‐Gómez 2018). Once the landscape of $\sum E_{a_{\mathrm{ij}}}^{\sigma^{2}}$ is mapped across environmental autocorrelation and variance, the relative contributions of constraints, phylogeny and species‐specific effects on $\sum E_{a_{\mathrm{ij}}}^{\sigma^{2}}$ will be realised. This combined approach of modelling and dimension reduction offers generalisation in a previously exception driven area of life history theory.

## Author Contributions

S.J.L.G., I.S. and R.S.‐G. conceived and managed the project. S.J.L.G., M.K., I.S. and R.S.‐G. provided early idea development. S.J.L.G., M.K., G.S., S.T. and R.S.‐G. contributed to early methods development. S.J.L.G. coded the simulation, performed the analysis and wrote the first draft with contributions from I.S. and R.S.‐G. Later idea contributions and edits to the manuscript came from S.T., A.C., U.K.S., A.C.V. and H.J. All authors contributed significantly to the final manuscript.

### Peer Review

The peer review history for this article is available at https://www.webofscience.com/api/gateway/wos/peer‐review/10.1111/ele.70066.

## Supporting information


Data S1.

## Data Availability

All data and code supporting these results are published on Zenodo at https://zenodo.org/records/14313230 and are also available on GitHub at https://github.com/SamuelGascoigne/Structured_Demographic_Buffering.

## References

[ele70066-bib-0001] Arthreya, K. B. , and S. Karlin . 1971. “On Branching Processes With Random Environments: I: Extinction Probabilities.” Annals of Mathematical Statistics 42: 1499–1520.

[ele70066-bib-0002] Barraquand, F. , and N. G. Yoccoz . 2013. “When Can Environmental Variability Benefit Population Growth? Counterintuitive Effects of Nonlinearities in Vital Rates.” Theoretical Population Biology 89: 1–11. 23906589 10.1016/j.tpb.2013.07.002

[ele70066-bib-0003] Bathiany, S. , V. Dakos , M. Scheffer , and T. M. Lenton . 2018. “Climate Models Predict Increasing Temperature Variability in Poor Countries.” Science Advances 4: 1–11. 10.1126/sciadv.aar5809 PMC5931768 29732409

[ele70066-bib-0004] Bruijning, M. , C. J. E. Metcalf , E. Jongejans , and J. F. Ayroles . 2020. “The Evolution of Variance Control.” Trends in Ecology & Evolution 35: 22–33. 31519463 10.1016/j.tree.2019.08.005 PMC7482585

[ele70066-bib-0005] Bull, J. C. , N. J. Pickup , B. Pickett , M. P. Hassell , and M. B. Bonsall . 2007. “Metapopulation Extinction Risk Is Increased by Environmental Stochasticity and Assemblage Complexity.” Proceedings of the Royal Society B: Biological Sciences 274: 87–96. 10.1098/rspb.2006.3691 PMC1679879 17018431

[ele70066-bib-0006] Capdevila, P. , I. Stott , M. Beger , and R. Salguero‐Gómez . 2020. “Towards a Comparative Framework of Demographic Resilience.” Trends in Ecology & Evolution 35: 776–786. 32482368 10.1016/j.tree.2020.05.001

[ele70066-bib-0007] Capdevila, P. , I. Stott , J. Cant , et al. 2022. “Life History Mediates the Trade‐Offs Among Different Components of Demographic Resilience.” Ecology Letters 25: 1566–1579. 35334148 10.1111/ele.14004 PMC9314072

[ele70066-bib-0008] Caswell, H. 2001. Matrix Population Models: Construction, Analysis, and Interpretation. 2nd ed. Sunderland, MA: Sinauer.

[ele70066-bib-0009] Dalgleish, H. J. , D. N. Koons , and P. B. Adler . 2010. “Can Life‐History Traits Predict the Response of Forb Populations to Changes in Climate Variability?” Journal of Ecology 98: 209–217.

[ele70066-bib-0010] de Roos, A. M. 1997. “A Gentle Introduction to Physiologically Structured Population Models.” In Structured‐Population Models in Marine, Terrestrial, and Freshwater Systems, edited by S. Tuljapurkar and H. Caswell , 119–204. Boston, MA: Springer.

[ele70066-bib-0011] Di Cecco, G. J. , and T. C. Gouhier . 2018. “Increased Spatial and Temporal Autocorrelation of Temperature Under Climate Change.” Scientific Reports 8: 1–9. 30287852 10.1038/s41598-018-33217-0 PMC6172201

[ele70066-bib-0012] Drake, J. M. 2005. “Population Effects of Increased Climate Variation.” Proceedings of the Royal Society B: Biological Sciences 272: 1823–1827. 10.1098/rspb.2005.3148 PMC1559868 16096095

[ele70066-bib-0013] Easterling, M. R. , S. P. Ellner , and P. M. Dixon . 2000. “Size‐Specific Sensitivity: Applying a New Structured Population Model.” Ecology 81: 694–708.

[ele70066-bib-0014] Ebert, T. A. 1999. Populations Methods in Demography. Methods & Demography. San Diego, CA: Academic Press.

[ele70066-bib-0015] Ellner, S. P. , D. Z. Childs , and M. Rees . 2016. Data‐Driven Modelling of Structured Populations. Cham: Springer International Publishing.

[ele70066-bib-0016] Engen, S. , Ø. Bakke , and A. Islam . 1998. “Demographic and Environmental Stochasticity‐Concepts and Definitions.” Biometrics 54: 840–846.

[ele70066-bib-0017] Evers, S. M. , T. M. Knight , and A. Compagnoni . 2023. “The Inclusion of Immediate and Lagged Climate Responses Amplifies the Effect of Climate Autocorrelation on Long‐Term Growth Rate of Populations.” Journal of Ecology 111: 1–12.

[ele70066-bib-0018] Foley, P. 1994. “Predicting Extinction Times From Environmental Stochasticity and Carrying Capacity.” Conservation Biology 8: 124–137.

[ele70066-bib-0019] Gaillard, J.‐M. , M. Festa‐Bianchet , and N. G. Yoccoz . 1998. “Population Dynamics of Large Herbivores: Variable Recruitment With Constant Adult Survival.” Trends in Ecology & Evolution 13: 249–251. 10.1016/s0169-5347(97)01237-8 21238201

[ele70066-bib-0020] Gascoigne, S. J. L. , M. Kajin , and R. Salguero‐Gómez . 2023. “Criteria for Buffering in Ecological Modeling.” Trends in Ecology & Evolution 39: 116–118. 38042645 10.1016/j.tree.2023.11.006

[ele70066-bib-0021] Gascoigne, S. J. L. , M. Kajin , I. Sepil , and R. Salguero‐Gómez . 2024. “Testing for Efficacy in Four Measures of Demographic Buffering.” *EcoEvoRxiv*. 10.32942/X23911.

[ele70066-bib-0022] Griffith, A. B. 2017. “Perturbation Approaches for Integral Projection Models.” Oikos 126: 1675–1686.

[ele70066-bib-0023] Halofsky, J. E. , D. L. Peterson , and B. J. Harvey . 2020. “Changing Wildfire, Changing Forests: The Effects of Climate Change on Fire Regimes and Vegetation in the Pacific Northwest, USA.” Fire Ecology 16: 4.

[ele70066-bib-0024] Haridas, C. V. , and S. Tuljapurkar . 2005. “Elasticities in Variable Environments: Properties and Implications.” American Naturalist 166: 481–495. 10.1086/444444 16224704

[ele70066-bib-0025] Healy, K. , T. H. G. Ezard , O. R. Jones , R. Salguero‐Gómez , and Y. M. Buckley . 2019. “Animal Life History Is Shaped by the Pace of Life and the Distribution of Age‐Specific Mortality and Reproduction.” Nature Ecology & Evolution 3: 1217–1224. 31285573 10.1038/s41559-019-0938-7

[ele70066-bib-0026] Higgins, S. I. , S. T. A. Pickett , and W. J. Bond . 2000. “Predicting Extinction Risks for Plants: Environmental Stochasticity Can Save Declining Populations.” Trends in Ecology & Evolution 15: 516–520. 11114439 10.1016/s0169-5347(00)01993-5

[ele70066-bib-0027] Hilde, C. H. , M. Gamelon , B. E. Sæther , J. M. Gaillard , N. G. Yoccoz , and C. Pélabon . 2020. “The Demographic Buffering Hypothesis: Evidence and Challenges.” Trends in Ecology & Evolution 35: 523–538. 32396819 10.1016/j.tree.2020.02.004

[ele70066-bib-0028] Hoffmann, A. A. , and J. Bridle . 2022. “Plasticity and the Costs of Incorrect Responses.” Trends in Ecology & Evolution 38: 219–220. 36528412 10.1016/j.tree.2022.11.012

[ele70066-bib-0029] Jackson, J. , C. Le Coeur , and O. Jones . 2022. “Life‐History Predicts Global Population Responses to the Weather in the Terrestrial Mammals.” eLife 11: e74161. 35775734 10.7554/eLife.74161 PMC9307275

[ele70066-bib-0030] Jensen, J. L. W. V. 1906. “Sur les fonctions convexes et les inégalités entre les valeurs moyennes.” Acta Mathematica 30: 175–193.

[ele70066-bib-0031] Jones, O. R. , A. Scheuerlein , R. Salguero‐Gómez , et al. 2014. “Diversity of Ageing Across the Tree of Life.” Nature 505: 169–173. 24317695 10.1038/nature12789 PMC4157354

[ele70066-bib-0032] Jongejans, E. , H. de Kroon , S. Tuljapurkar , and K. Shea . 2010. “Plant Populations Track Rather Than Buffer Climate Fluctuations.” Ecology Letters 13: 736–743. 20426793 10.1111/j.1461-0248.2010.01470.x

[ele70066-bib-0033] King, J. G. , and J. D. Hadfield . 2019. “The Evolution of Phenotypic Plasticity When Environments Fluctuate in Time and Space.” Evolution Letters 3: 15–27. 30788139 10.1002/evl3.100 PMC6369965

[ele70066-bib-0034] Klinkhammer, P. G. L. , and T. J. de Jong . 1983. “Is It Profitable for Biennials to Live Longer Than Two Years.” Ecological Modelling 20: 223–232.

[ele70066-bib-0035] Knutson, T. R. , J. L. McBride , J. Chan , et al. 2010. “Tropical Cyclones and Climate Change.” Nature Geoscience 3: 157–163.

[ele70066-bib-0036] Koons, D. N. , S. Pavard , A. Baudisch , and C. J. E. Metcalf . 2009. “Is Life‐History Buffering or Lability Adaptive in Stochastic Environments?” Oikos 118: 972–980.

[ele70066-bib-0037] Lande, R. , B. E. Sæther , and S. Engen . 1997. “Threshold Harvesting for Sustainability of Fluctuating Resources.” Ecology 78: 1341–1350.

[ele70066-bib-0038] Le Coeur, C. , N. G. Yoccoz , R. Salguero‐Gómez , and Y. Vindenes . 2022. “Life History Adaptations to Fluctuating Environments: Combined Effects of Demographic Buffering and Lability of Demographic Parameters.” Ecology Letters 25: 1–13. 10.1111/ele.14071 PMC9804727 35986627

[ele70066-bib-0039] Lennartsson, T. , and J. G. B. Oostermeijer . 2001. “Demographic Variation and Population Viability in Gentianella Campestris: Effects of Grassland Management and Environmental Stochasticity.” Journal of Ecology 89: 451–463.

[ele70066-bib-0040] Levin, S. C. , S. Evers , T. Potter , et al. 2022. “Rpadrino: An R Package to Access and Use PADRINO, an Open Access Database of Integral Projection Models.” Methods in Ecology and Evolution 2022: 1–7.

[ele70066-bib-0041] Lewis, S. C. , and A. D. King . 2017. “Evolution of Mean, Variance and Extremes in 21st Century Temperatures.” Weather and Climate Extremes 15: 1–10.

[ele70066-bib-0042] Mack, R. N. 2000. “Cultivation Fosters Plant Naturalization by Reducing Environmental Stochasticity.” Biological Invasions 2: 111–122.

[ele70066-bib-0043] Marques, G. M. , S. Augustine , K. Lika , L. Pecquerie , T. Domingos , and S. A. L. M. Kooijman . 2018. “The AmP Project: Comparing Species on the Basis of Dynamic Energy Budget Parameters.” PLoS Computational Biology 14: e1006100. 29742099 10.1371/journal.pcbi.1006100 PMC5962104

[ele70066-bib-0044] Masson‐Delmotte, V. , P. Zhai , A. Pirani , et al. 2021. IPCC: Climate Change 2021: The Physical Science Basis. Cambridge: Cambridge University Press.

[ele70066-bib-0045] May, R. M. 1973. “Stability in Randomly Fluctuating Versus Deterministic Environments.” American Naturalist 107: 621–650.

[ele70066-bib-0046] McDonald, J. L. , M. Franco , S. Townley , T. H. G. Ezard , K. Jelbert , and D. J. Hodgson . 2017. “Divergent Demographic Strategies of Plants in Variable Environments.” Nature Ecology & Evolution 1: 0029. 10.1038/s41559-016-0029 28812611

[ele70066-bib-0047] McDonald, J. L. , I. Stott , S. Townley , and D. J. Hodgson . 2016. “Transients Drive the Demographic Dynamics of Plant Populations in Variable Environments.” Journal of Ecology 104: 306–314. 26973355 10.1111/1365-2745.12528 PMC4768644

[ele70066-bib-0048] Melbourne, B. A. , and A. Hastings . 2008. “Extinction Risk Depends Strongly on Factors Contributing to Stochasticity.” Nature 454: 100–103. 18596809 10.1038/nature06922

[ele70066-bib-0049] Merow, C. , S. T. Bois , J. M. Allen , Y. Xie , and J. A. Silander . 2017. “Climate Change Both Facilitates and Inhibits Invasive Plant Ranges in New England.” Proceedings of the National Academy of Sciences of the United States of America 114: E3276–E3284. 28348212 10.1073/pnas.1609633114 PMC5402445

[ele70066-bib-0050] Morris, W. F. , and D. F. Doak . 2004. “Buffering of Life Histories Against Environmental Stochasticity: Accounting for a Spurious Correlation Between the Variabilities of Vital Rates and Their Contributions to Fitness.” American Naturalist 163: 579–590. 10.1086/382550 15122504

[ele70066-bib-0051] Morris, W. F. , C. A. Pfister , S. Tuljapurkar , et al. 2008. “Longevity Can Buffer Plant and Animal Populations Against Changing Climatic Variability.” Ecology 89: 19–25. 18376542 10.1890/07-0774.1

[ele70066-bib-0052] Murren, C. J. , H. J. Maclean , S. E. Diamond , et al. 2014. “Evolutionary Change in Continuous Reaction Norms.” American Naturalist 183: 453–467. 10.1086/675302 24642491

[ele70066-bib-0053] Neubert, M. G. , and H. Caswell . 1997. “Alternatives to Resilience for Measuring the Responses of Ecological Systems to Perturbations.” Ecology 78: 653–665.

[ele70066-bib-0054] Nisbet, R. M. , E. B. Muller , K. Lika , and S. A. L. M. Kooijman . 2000. “From Molecules to Ecosystems Through Dynamic Energy Budget Models.” Journal of Animal Ecology 69: 913–926.

[ele70066-bib-0055] Orzack, S. H. 1985. “Population Dynamics in Variable Environments. V. The Genetics of Homeostasis Revisited.” American Naturalist 125: 550–572.

[ele70066-bib-0056] Orzack, S. H. , and S. Tuljapurkar . 1989. “Population Dynamics in Variable Environments. VII. The Demography and Evolution of Iteroparity.” American Naturalist 133: 901–923.

[ele70066-bib-0057] Paniw, M. , A. Ozgul , and R. Salguero‐Gómez . 2018. “Interactive Life‐History Traits Predict Sensitivity of Plants and Animals to Temporal Autocorrelation.” Ecology Letters 21: 275–286. 29266843 10.1111/ele.12892

[ele70066-bib-0058] Petchey, O. L. 2000. “Environmental Colour Affects Aspects of Single‐Species Population Dynamics.” Proceedings of the Royal Society B: Biological Sciences 267: 747–754. 10.1098/rspb.2000.1066 PMC1690600 10819142

[ele70066-bib-0059] Petchey, O. L. , A. Gonzalez , and H. B. Wilson . 1997. “Effects on Population Persistence: The Interaction Between Environmental Noise Colour, Intraspecific Competition and Space.” Proceedings of the Royal Society of London: Biological Sciences 264: 1841–1847.

[ele70066-bib-0060] Pfister, C. A. 1998. “Patterns of Variance in Stage‐Structured Populations: Evolutionary Predictions and Ecological Implications.” Proceedings of the National Academy of Sciences of the United States of America 95: 213–218. 9419355 10.1073/pnas.95.1.213 PMC34554

[ele70066-bib-0061] Rademaker, M. , A. van Leeuwen , and I. M. Smallegange . 2024. “Why We Cannot Always Expect Life History Strategies to Directly Inform on Sensitivity to Environmental Change.” Journal of Animal Ecology 93: 348–366. 38303132 10.1111/1365-2656.14050

[ele70066-bib-0062] Revell, L. J. 2012. “Phytools: An R Package for Phylogenetic Comparative Biology (And Other Things).” Methods in Ecology and Evolution 3: 217–223.

[ele70066-bib-0063] Roerdink, J. B. T. M. 1988. “The Biennial Life Strategy in a Random Environment.” Journal of Mathematical Biology 26: 199–215.

[ele70066-bib-0064] Roerdink, J. B. T. M. 1989. “The Biennial Life Strategy in a Random Environment: Supplement.” Journal of Mathematical Biology 27: 309–319.

[ele70066-bib-0065] Romeijn, J. , and I. M. Smallegange . 2022. “Exploring How the Fast‐Slow Pace of Life Continuum and Reproductive Strategies Structure Microorganism Life History Variation.” *bioRxiv*. 10.1101/2022.11.28.517963.

[ele70066-bib-0066] Roughgarden, J. 1975. “A Simple Model for Population Dynamics in Stochastic Environments.” American Naturalist 109: 713–736.

[ele70066-bib-0067] Sæther, B. E. , T. Coulson , V. Grøtan , et al. 2013. “How Life History Influences Population Dynamics in Fluctuating Environments.” American Naturalist 182: 743–759. 10.1086/673497 24231536

[ele70066-bib-0068] Saether, E. , M. C. Islam , and C. Perrins . 1998. “Environmental Stochasticity and Extinction Risk in a Population of a Small Songbird, the Great Tit.” American Naturalist 151: 441–450. 10.1086/286131 18811318

[ele70066-bib-0069] Salguero‐Gómez, R. , O. R. Jones , C. R. Archer , et al. 2016. “COMADRE: A Global Data Base of Animal Demography.” Journal of Animal Ecology 85: 371–384. 26814420 10.1111/1365-2656.12482 PMC4819704

[ele70066-bib-0070] Salguero‐Gómez, R. , O. R. Jones , C. R. Archer , et al. 2015. “The Compadre Plant Matrix Database: An Open Online Repository for Plant Demography.” Journal of Ecology 103: 202–218.

[ele70066-bib-0071] Salguero‐Gómez, R. , O. R. Jones , E. Jongejans , et al. 2016. “Fast–Slow Continuum and Reproductive Strategies Structure Plant Life‐History Variation Worldwide.” Proceedings of the National Academy of Sciences of the United States of America 113: 230–235. 26699477 10.1073/pnas.1506215112 PMC4711876

[ele70066-bib-0072] Santos, G. S. , S. J. L. Gascoigne , A. T. C. Dias , M. Kajin , and R. Salguero‐Gómez . 2023. “A Unified Framework to Identify Demographic Buffering in Natural Populations.” *bioRxiv*, 1–31. 10.1101/2023.07.03.547528. PMC13439591 41631607

[ele70066-bib-0073] Shen, S. S. P. , A. B. Gurung , H. S. Oh , T. Shu , and D. R. Easterling . 2011. “The Twentieth Century Contiguous US Temperature Changes Indicated by Daily Data and Higher Statistical Moments.” Climatic Change 109: 287–317.

[ele70066-bib-0074] Sheth, S. N. , and A. L. Angert . 2018. “Demographic Compensation Does Not Rescue Populations at a Trailing Range Edge.” Proceedings of the National Academy of Sciences of the United States of America 115: 2413–2418. 29463711 10.1073/pnas.1715899115 PMC5878003

[ele70066-bib-0075] Smallegange, I. M. , H. Caswell , M. E. M. Toorians , and A. M. de Roos . 2017. “Mechanistic Description of Population Dynamics Using Dynamic Energy Budget Theory Incorporated Into Integral Projection Models.” Methods in Ecology and Evolution 8: 146–154.

[ele70066-bib-0076] Smallegange, I. M. , J. A. Deere , and T. Coulson . 2014. “Correlative Changes in Life‐History Variables in Response to Environmental Change in a Model Organism.” American Naturalist 183: 784–797. 10.1086/675817 24823822

[ele70066-bib-0077] Stott, I. , S. Townley , and D. J. Hodgson . 2011. “A Framework for Studying Transient Dynamics of Population Projection Matrix Models.” Ecology Letters 14: 959–970. 21790932 10.1111/j.1461-0248.2011.01659.x

[ele70066-bib-0078] Sutherland, W. J. , R. P. Freckleton , H. C. J. Godfray , et al. 2013. “Identification of 100 Fundamental Ecological Questions.” Journal of Ecology 101: 58–67.

[ele70066-bib-0079] Trenberth, K. E. 2011. “Changes in Precipitation With Climate Change.” Climate Research 47: 123–138.

[ele70066-bib-0080] Tuljapurkar, S. 1982. “Population Dynamics in Variable Environments. III. Evolutionary Dynamics of r‐Selection.” Theoretical Population Biology 21: 141–165. 10.1016/0040-5809(85)90019-x 4060082

[ele70066-bib-0081] Tuljapurkar, S. 1989. “An Uncertain Life: Demography in Random Environments.” Theoretical Population Biology 35: 227–294. 2756495 10.1016/0040-5809(89)90001-4

[ele70066-bib-0082] Tuljapurkar, S. 1990. Population Dynamics in Variable Environments. Berlin, Heidelberg: Springer‐Verlag.

[ele70066-bib-0083] Tuljapurkar, S. , J. M. Gaillard , and T. Coulson . 2009. “From Stochastic Environments to Life Histories and Back.” Philosophical Transactions of the Royal Society, B: Biological Sciences 364: 1499–1509. 10.1098/rstb.2009.0021 PMC2690509 19414465

[ele70066-bib-0084] Tuljapurkar, S. , and C. V. Haridas . 2006. “Temporal Autocorrelation and Stochastic Population Growth.” Ecology Letters 9: 327–337. 16958899 10.1111/j.1461-0248.2006.00881.x

[ele70066-bib-0085] Tuljapurkar, S. , C. C. Horvitz , and J. B. Pascarella . 2003. “The Many Growth Rates and Elasticities of Populations in Random Environments.” American Naturalist 162: 489–502. 10.1086/378648 14582010

[ele70066-bib-0086] Tuljapurkar, S. , and C. Istock . 1993. “Environmental Uncertainty and Variable Diapause.” Theoretical Population Biology 43: 251–280. 8327984 10.1006/tpbi.1993.1011

[ele70066-bib-0087] Tuljapurkar, S. , H. Jaggi , S. J. L. Gascoigne , W. Zuo , M. Kajin , and R. Salguero‐Gómez . 2023. “From Disturbances to Nonlinear Fitness and Back.” *bioRxiv*. 10.1101/2023.10.20.563360.

[ele70066-bib-0088] Tuljapurkar, S. , and R. Lee . 1997. “Demographic Uncertainty and the Stable Equivalent Population.” Mathematical and Computer Modelling 26: 39–56.

[ele70066-bib-0089] Urban, M. C. 2015. “Accelerating Extinction Risk From Climate Change.” Science 1979, no. 348: 571–573. 10.1126/science.aaa4984 25931559

[ele70066-bib-0090] Varas‐Enriquez, P. J. , S. van Daalen , and H. Caswell . 2022. “Individual Stochasticity in the Life History Strategies of Animals and Plants.” *PLoS One. 17: e0273407*. 10.1371/journal.pone.0273407 PMC9506618 36149850

[ele70066-bib-0091] Vinton, A. C. , S. J. L. Gascoigne , I. Sepil , and R. Salguero‐Gómez . 2022. “Plasticity's Role in Adaptive Evolution Depends on Environmental Change Components.” Trends in Ecology & Evolution 37: 1067–1078. 36153155 10.1016/j.tree.2022.08.008

[ele70066-bib-0092] Vinton, A. C. , S. J. L. Gascoigne , I. Sepil , and R. Salguero‐Gómez . 2023. “The Importance of Spatial and Temporal Structure in Determining the Interplay Between Plasticity and Evolution.” Trends in Ecology & Evolution 38: 221–223. 36610919 10.1016/j.tree.2022.12.009

[ele70066-bib-0093] Wang, J. , X. Yang , G. Silva Santos , et al. 2023. “Flexible Demographic Strategies Promote the Population Persistence of a Pioneer Conifer Tree ( *Pinus massoniana* ) in Ecological Restoration.” Forest Ecology and Management 529: 120727.

[ele70066-bib-0094] Westerband, A. C. , and C. C. Horvitz . 2017. “Photosynthetic Rates Influence the Population Dynamics of Understory Herbs in Stochastic Light Environments.” Ecology 98: 370–381. 27870009 10.1002/ecy.1664

[ele70066-bib-0095] Wieczynski, D. J. , P. E. Turner , and D. A. Vasseur . 2018. “Temporally Autocorrelated Environmental Fluctuations Inhibit the Evolution of Stress Tolerance.” American Naturalist 191: E195–E207. 10.1086/697200 29750560

[ele70066-bib-0096] Wiener, P. , and S. Tuljapurkar . 1994. “Migration in Variable Environments: Exploring Life‐History Evolution Using Structured Population Models.” Journal of Theoretical Biology 166: 75–90. 8145562 10.1006/jtbi.1994.1006

