# Supplementary material for: Structured Demographic Buffering: A Framework to Explore the Environmental Components and Demographic Mechanisms Underlying Demographic Buffering
Source: Ecol Lett. 2025 Feb 25;28(2):e70066. doi: 10.1111/ele.70066 (PMC11862329; doi:10.1111/ele.70066)
Supplement: Supplementary file 1 — Data S1. [file ELE-28-0-s001.pdf]

Structured demographic buffering: A framework to explore the environment drivers and demographic mechanisms underlying demographic buffering

Supplementary online materials

Samuel J L Gascoigne[1,2,*], Maja Kajin[1,3], Shripad Tuljapurkar[4], Gabriel Silva Santos[5], Aldo Compagnoni[6,7], Ulrich K Steiner[8], Anna C Vinton[1], Harman Jaggi[4], Irem Sepil[1] & Roberto Salguero-Gómez [1,9]

[1] Department of Biology, South Parks Road, University of Oxford, Oxford, United Kingdom
[2] School of Biological Sciences, University of Aberdeen, Aberdeen, United Kingdom
[3] Department of Biology, Biotechnical Faculty, University of Ljubljana, Večna pot 111, 1000 Ljubljana, Slovenia
[4] Biology Department, Stanford University, Stanford, CA, USA
[5] National Institute of the Atlantic Forest (INMA), Santa Teresa, Espírito Santo, Brazil
[6] Institute of Biology, Martin Luther University Halle-Wittenburg, Halle (Saale), Germany
[7] German Centre for Integrative Biodiversity Research (iDiv) Halle-Jena-Leipzig, Leipzig, Germany
[8] Institute of Biology, Freie Universität Berlin, Berlin, Germany
[9] National Laboratory for Grassland & Agro-ecosystems, Lanzhou University, China

***** corresponding e-mail: samuel.gascoigne@abdn.ac.uk

Table of Contents

Supplementary Figure 1. Distribution of λs from simulations across the environment autocorrelation – variance parameter space. 3

Supplementary Table 1. Formulas, regressions and parameters used to construct the IPMs for *Berberis thunbergii*. 4

Supplementary Table 2. Formulas, regressions and parameters used to construct the IPMs for *Calathea crotalifera*. 6

Supplementary Table 3. Formulas, regressions and parameters used to construct the IPMs for *Heliconia tortuosa*. 8

Standardised analysis pipeline. 10

Supplementary Table 4. Model selection to quantify the effects of environmental autocorrelation and variance on demographic buffering (DB) in *Berberis thunbergii*. 11

Supplementary Table 5. Model selection to quantify the effects of environmental autocorrelation and variance on demographic buffering (DB) in *Calathea crotalifera*. 12

Supplementary Table 6. Model selection to quantify the effects of environmental autocorrelation and variance on demographic buffering (DB) in *Heliconia tortuosa*. 13

Supplementary Table 7. Model selection to quantify the effects of environmental autocorrelation and variance on the differences of 14

Supplementary Table 8. Model selection to quantify the effects of environmental autocorrelation and variance on the differences of 15

Supplementary Table 9. Model selection to quantify the effects of environmental autocorrelation and variance on the differences of 16

Supplementary Table 10. Model selection to quantify the effects of environmental autocorrelation and variance on the mean buffered size in *Berberis thunbergii* 17

Supplementary Table 11. Model selection to quantify the effects of environmental autocorrelation and variance on the mean buffered size in *Calathea crotalifera*. 18

Supplementary Table 12. Model selection to quantify the effects of environmental autocorrelation and variance on the mean buffered size in *Heliconia tortuosa*. 19

Supplementary Table 13. Model selection to quantify the effects of environmental autocorrelation and variance on the **P-F** contribution in *Berberis thunbergii*. 20

Supplementary Table 14. Model selection to quantify the effects of environmental autocorrelation and variance on the **P-F** contribution in *Calathea crotalifera*. 21

Supplementary Table 15. Model selection to quantify the effects of environmental autocorrelation and variance on the **P-F** contribution in *Heliconia tortuosa* 22

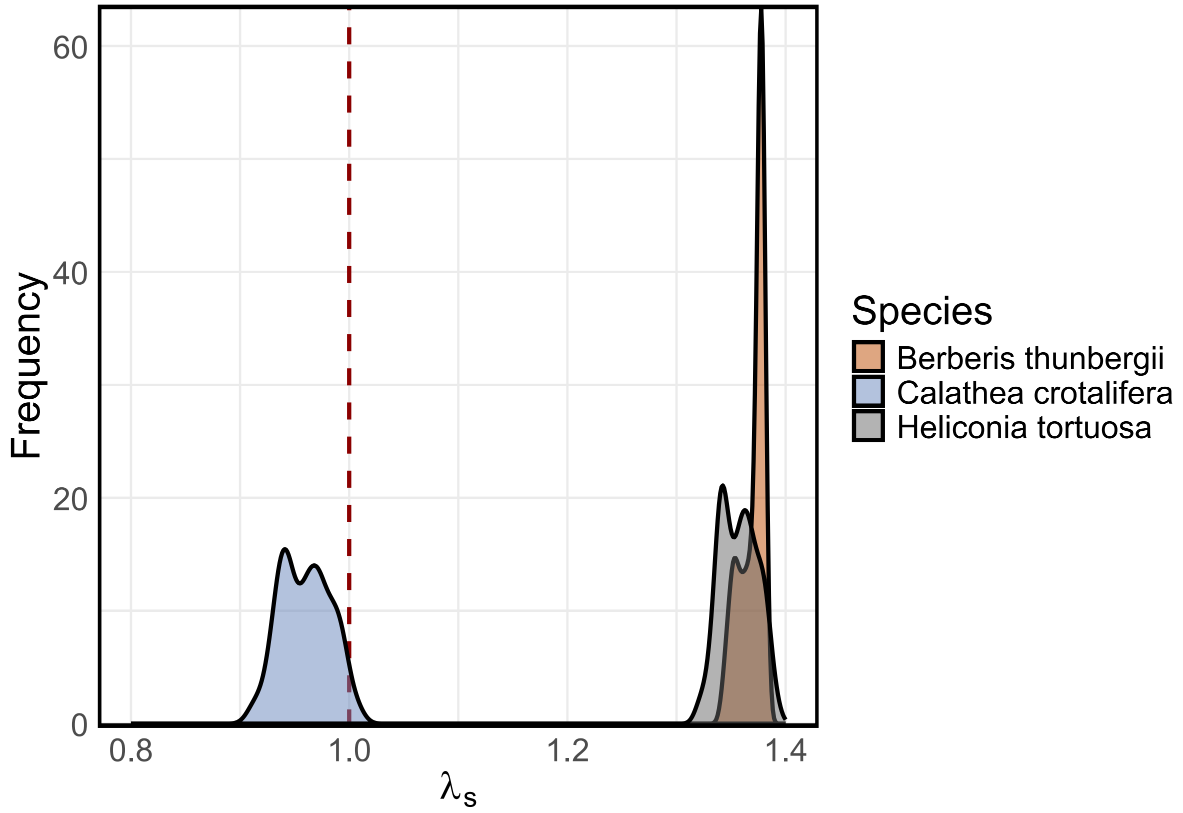

Supplementary Figure 1. Distribution of λ_s from simulations across the environment autocorrelation – variance parameter space. The red dashed line represents a population that stays stable when projected across time steps (*i.e.*, $\lambda_s = 1$). Values distributed to the left of the red dashed line represent populations who will asymptotically decline in number across time steps (*i.e.*, $\lambda_s < 1$), whilst values distributed to the right of the red dashed line represent populations that will asymptotically increase across time steps (*i.e.*, $\lambda_s > 1$).

Supplementary Table 1. Formulas, regressions and parameters used to construct the IPMs for *Berberis thunbergii*.

Construction		Model	Parameter
Density-independent environmentally stochastic IPM		$n(z', t+1) = \int_{\alpha}^{\omega} K(z', z, \psi_t) n(z, t) dz$	α = 2<br>ω = 25<br>z = log(plant area)
		$\psi_t = \{T_t, P_t, PAR_t, N_t, pH_t\}$	ψ = an array containing climate values
K-kernel		$K(z', z, \psi_t) = P(z', z, \psi_t) + F(z', z, \psi_t)$	
Sub-kernels	***P***-subkernel	$P(z', z, \psi_t) = s(z, \psi_t) * g(z', z, \psi_t)$	
	F-subkernel	$F(z', z, \psi_t) = f_s(z) * fl_p(z) * germ_p(\psi_t) * sdl_s(z')$	
Demographic functions	Survival	$\text{logit}(s(z, \psi_t)) = s_i + s_z * z + s_T * T_t + s_P * P_t + s_{PAR} * PAR_t + s_N * N_t + s_{pH} * pH_t$	s_i = -11.8<br>s_z = 1.05<br>s_T = 1.11<br>s_P = 0.22<br>s_{PAR} = -0.52<br>s_N = -0.1<br>s_{pH} = 0.11
	Growth	$g(z', z, \psi_t) = \text{dnorm}(z', g_{\mu}(z, \psi_t), g_{sd})$	g_{sd} = 1.48
		$g_{\mu}(z, \psi_t) = g_z * z + g_T * T_t + g_P * P_t + g_{PAR} * PAR_t + g_N * N_t + g_{pH} * pH_t$	g_z = 1.02<br>g_T = 0.65<br>g_P = 0.02<br>g_{PAR} = 0.59<br>g_N = -0.04<br>g_{pH} = 0.4
	Reproduction	$f_s(z) = \exp(seed_i + seed_z * z)$	$seed_i$ = -23.01<br>$seed_z$ = 1.32
		$\text{logit}(fl_p(z)) = fl_i + fl_z * z$	fl_i = -33.43<br>fl_z = 1.68

		$\text{logit}(germ_p(\psi_t)) = germ_i + germ_T * T_t + germ_P * P_t + germ_{PAR} * (PAR_t/0.018) + germ_{pH} * pH_t$	$germ_i$ = -11.8<br>$germ_T$ = 0.51<br>$germ_P$ = -0.02<br>$germ_{PAR}$ = -0.02<br>$germ_{pH}$ = 0.26
		$sdl_s(z') = \text{dnorm}(z', sdl_\mu, sdl_{sd})$	sdl_μ = 10.23<br>sdl_{sd} = 1.581
Environment values	Mean temperature in warmest month	$T \sim \text{N}(0, 1.5)$	
	Mean May precipitation	$P \sim \text{N}(0, 1.5)$	
	PAR	$PAR \sim \text{N}(0, 1.5)$	
	Soil Nitrogen	$N \sim \text{N}(0, 1.5)$	
	Soil pH	$pH \sim \text{N}(0, 1.5)$	

Supplementary Table 2. Formulas, regressions and parameters used to construct the IPMs for *Calathea crotalifera*.

Construction		Model	Parameter
Density-independent environmentally stochastic IPM		$n(z',t+1) = \int_{\alpha}^{\omega} K(z',z,\psi_t)n(z,t)dz$	α = 0.57<br>ω = 11.9<br>z = leaf area
		$\psi_t = \{j_t, A_t\}$	ψ = an array containing climate values
***K*-kernel**		$K(z',z,\psi_t) = P(z',z,j_t,A_t) + F(z',z,j_t)$	
Sub-kernels	***P*-kernel**	$P(z',z,j_t,A_t) = s(z,j_t) * g(z',z,j_t,A_t)$	
	***F*-kernel**	$F(z',z,j_t) = r_p(z,j_t) * r_o(z,j_t) * n_f * n_s * s_s(j_t) * sdl_s(j_t) * sdl_{size}(z',j_t)$	n_f = 23<br>n_s = 3
Demographic functions	Survival	$\text{logit}(s(z,\psi_t)) = s_i + s_z * z + s_j * j_t + s_{z*j} * z * j_t$	s_i = -2.74<br>s_z = 0.95<br>s_j = 0.07<br>s_{z*j} = -0.02
	Growth	$g(z',z,j_t,A_t) = \text{dnorm}(z', g_\mu(z,j_t,A_t), g_{sd})$	g_{sd} = 1.53
		$g_\mu(z,j_t,A_t) = g_i + g_z * z + g_j * j_t + g_A * A_t + g_{z*j} * z * j_t + g_{z*A} * z * A_t + g_{j*A} * j_t * A_t + g_{z*j*A} * z * j_t * A_t$	g_i = 0.76<br>g_z = 0.9<br>g_j = 0.03<br>g_A = 0.006<br>g_{z*j} = -0.001<br>g_{z*A} = 0.00045<br>g_{j*A} = -0.0052<br>g_{z*j*A} = 0.00035
	Reproduction	$\text{logit}(r_p(z,j_t)) = r_{p,i} + r_{p,z} * z + r_{p,j} * j_t + r_{p,z*j} * z * j_t$	$r_{p,i}$ = -13.23<br>$r_{p,z}$ = 1.401<br>$r_{p,j}$ = -0.213<br>$r_{p,z*j}$ = 0.043
		$r_o(z,j_t) = \exp(r_{o,i} + r_{o,z} * z + r_{o,j} * j_t + r_{o,z*j} * z * j_t)$	$r_{o,i}$ = -6.673<br>$r_{o,z}$ = 0.829

			$r_{o,j}$ = 0.067<br>$r_{o,z*j}$ = -0.007
		$s_s(j_t < 6) = 0.29$<br>$s_s(j_t \geq 6) = 0.32$	
		$sdl_s(j_t < 6) = 0.14$<br>$sdl_s(j_t \geq 6) = 0.95$	
		$sdl_{size}(z', j_t < 6) = \text{dnorm}(z', 3.08, 0.54)$<br>$sdl_{size}(z', j_t \geq 6) = \text{dnorm}(z', 2.88, 1.4)$	
Environment values	Canopy openness*	$j \sim \text{N}(3, 1.4)$	
	Photosynthetic rate*	$A \sim \text{N}(6, 0.8)$	

* In Westerband and Horvitz (2016), canopy openness (j) and photosynthetic rate (A) were modelled as random samples from a sequence of values or draws from a uniform distribution. Specifically canopy openness was realized at time t as random draws from the sequence $\{1, 2, 3, 4, 5\}$ whilst photosynthetic rate was realized at time t as random draws from a uniform distruction (*i.e.*, $A \sim \text{U}(5, 7)$). However, since our manipulation of the environment involves explicitly changing the temporal variance of a series, we coerced the distributions into normal distributions with the same mean and reported variance of the original sampling distributions reported in Westerband and Horvitz (2016).

Supplementary Table 3. Formulas, regressions and parameters used to construct the IPMs for *Heliconia tortuosa*.

Construction		Model	Parameter
Density-independent environmentally stochastic IPM		$n(z',t+1) = \int_{\alpha}^{\omega} K(z',z,\psi_t)n(z,t)dz$	α = 0.78<br>ω = 11.07<br>z = leaf area
		$\psi_t = \{j_t, A_t\}$	ψ = an array containing climate values
K-kernel		$K(z',z,\psi_t) = P(z',z,j_t,A_t) + F(z',z,j_t)$	
Sub-kernels	***P***-kernel	$P(z',z,j_t,A_t) = s(z,j_t) * g(z',z,j_t,A_t)$	
	F-kernel	$F(z',z,j_t) = r_p(z,j_t) * r_o(z,j_t) * n_f * n_s * s_s(j_t) * sdl_s(j_t) * sdl_{size}(z',j_t)$	n_f = 37<br>n_s = 2.5
Demographic functions	Survival	$\text{logit}\big(s(z,\psi_t)\big) = s_i + s_z * z + s_j * j_t + s_{z*j} * z * j_t$	s_i = -2.05<br>s_z = 0.78<br>s_j = -0.22<br>s_{z*j} = 0.05
	Growth	$g(z',z,j_t,A_t) = \text{dnorm}\big(z', g_\mu(z,j_t,A_t), g_{sd}\big)$	g_{sd} = 0.71
		$g_\mu(z,j_t,A_t) = g_i + g_z * z + g_j * j_t + g_A * A_t + g_{z*j} * z * j_t + g_{z*A} * z * A_t + g_{j*A} * j_t * A_t + g_{z*j*A} * z * j_t * A_t$	g_i = 2.6<br>g_z = 0.56<br>g_j = -1.55<br>g_A = 0.44<br>g_{z*j} = 0.18<br>g_{z*A} = -0.034<br>g_{j*A} = 0.014<br>g_{z*j*A} = -0.0014
	Reproduction	$\text{logit}(r_p(z,j_t)) = r_{p,i} + r_{p,z} * z + r_{p,j} * j_t + r_{p,z*j} * z * j_t$	$r_{p,i}$ = -12.55<br>$r_{p,z}$ = 1.527<br>$r_{p,j}$ = 0.154<br>$r_{p,z*j}$ = -0.013
		$r_o(z,j_t) = \exp(r_{o,i} + r_{o,z} * z + r_{o,j} * j_t + r_{o,z*j} * z * j_t)$	$r_{o,i}$ = -1.009<br>$r_{o,z}$ = 0.157

			$r_{o,j}$ = -0.382<br>$r_{o,z*j}$ = 0.048
		$s_s(j_t < 6) = 0.15$<br>$s_s(j_t \geq 6) = 0.2$	
		$sdl_s(j_t < 6) = 0.26$<br>$sdl_s(j_t \geq 6) = 0.33$	
		$sdl_{size}(z', j_t < 6) = \text{dnorm}(z', 2.73, 0.71)$<br>$sdl_{size}(z', j_t \geq 6) = \text{dnorm}(z', 2.34, 1.17)$	
Environment values	Canopy openness	$j \sim \text{N}(3, 1.4)$	
	Photosynthetic rate	$A \sim \text{N}(6.5, 0.8654937)$	

* In Westerband and Horvitz (2016), canopy openness (j) and photosynthetic rate (A) were modelled as random samples from a sequence of values or draws from a uniform distribution. Specifically canopy openness was realized at time t as random draws from the sequence $\{1, 2, 3, 4, 5\}$ whilst photosynthetic rate was realized at time t as random draws from a uniform distruction (*i.e.*, $A \sim \text{U}(5, 8)$). However, since our manipulation of the environment involves explicitly changing the temporal variance of a series, we coerced the distributions into normal distributions with the same mean and reported variance of the original sampling distributions reported in Westerband and Horvitz (2016).

Standardised analysis pipeline.

To investigate the roles of environmental autocorrelation and variance on demographic buffering, we used a standardised analysis pipeline to reproducibly model the linear and non-linear impacts of environment components (*i.e.*, environment autocorrelation and variance).

The standardised analysis pipeline has two phases. First, a suite of pre-defined statistical models were used to model the specified response variable (*e.g.*, **P**-**F** contribution). These models were classified as *a priori* (see below).

A priori models:

1. $response\ variable \sim \sigma^2 + \varphi + \sigma^2 * \varphi$
2. $response\ variable \sim \sigma^2 + \varphi + \sigma^2 * \varphi + \varphi^2$
3. $response\ variable \sim \sigma^2 + \varphi + \sigma^2 * \varphi + \varphi^2 + \varphi^3$
4. $response\ variable \sim \sigma^2 + \varphi + \sigma^2 * \varphi + (\sigma^2)^2$
5. $response\ variable \sim \sigma^2 + \varphi + \sigma^2 * \varphi + (\sigma^2)^2 + \varphi^2$
6. $response\ variable \sim \sigma^2 + \varphi + \sigma^2 * \varphi + (\sigma^2)^2 + \varphi^2 + \varphi^3$
7. $response\ variable \sim \sigma^2 + \varphi + \sigma^2 * \varphi + (\sigma^2)^2 + (\sigma^2)^3$
8. $response\ variable \sim \sigma^2 + \varphi + \sigma^2 * \varphi + (\sigma^2)^2 + (\sigma^2)^3 + \varphi^2$
9. $response\ variable \sim \sigma^2 + \varphi + \sigma^2 * \varphi + (\sigma^2)^2 + (\sigma^2)^3 + \varphi^2 + \varphi^3$

We chose this list of statistical models as they (1) contain linear and linear-interaction terms of environment autocorrelation (φ) and environment variance (σ^2) (see the first three terms of each model), (2) they sequentially add combinations of environment autocorrelation and variance up to a cubic term and (3) simplifies the combination of possible models generate from 7 predictors (127 possible combinations).

Supplementary Table 4. Model selection to quantify the effects of environmental autocorrelation and variance on demographic buffering (DB) in *Berberis thunbergii*. This model selection corresponds to the data show in Figure 2A in the main text. Environmental autocorrelation is denoted as φ whilst environmental variance is denoted as σ^2.

Model type	Model number	Model	DF	AIC	Selection
A priori	1	$DB \sim \sigma^2 + \varphi + \sigma^2 * \varphi$	5	-1333.377	
	2	$DB \sim \sigma^2 + \varphi + \sigma^2 * \varphi + \varphi^2$	6	-1566.159	
	3	$DB \sim \sigma^2 + \varphi + \sigma^2 * \varphi + \varphi^2 + \varphi^3$	7	-1681.584	
	4	$DB \sim \sigma^2 + \varphi + \sigma^2 * \varphi + (\sigma^2)^2$	6	-1349.455	
	5	$DB \sim \sigma^2 + \varphi + \sigma^2 * \varphi + (\sigma^2)^2 + \varphi^2$	7	-1619.828	
	6	$DB \sim \sigma^2 + \varphi + \sigma^2 * \varphi + (\sigma^2)^2 + \varphi^2 + \varphi^3$	8	-1783.319	**SELECTED**
	7	$DB \sim \sigma^2 + \varphi + \sigma^2 * \varphi + (\sigma^2)^2 + (\sigma^2)^3$	7	-1347.510	
	8	$DB \sim \sigma^2 + \varphi + \sigma^2 * \varphi + (\sigma^2)^2 + (\sigma^2)^3 + \varphi^2$	8	-1618.012	
	9	$DB \sim \sigma^2 + \varphi + \sigma^2 * \varphi + (\sigma^2)^2 + (\sigma^2)^3 + \varphi^2 + \varphi^3$	9	-1781.704	
Complete formula		$DB \sim -0.48626 + 1.2427383 * [\sigma^2] + 0.0004573 * [\varphi] + 0.0256003 * [\sigma^2 * \varphi] - 1.0036968 * [(\sigma^2)^2] - 0.0454271 * [\varphi^2] - 0.0501562 * [\varphi^3]$			

Supplementary Table 5. Model selection to quantify the effects of environmental autocorrelation and variance on demographic buffering (DB) in *Calathea crotalifera*. This model selection corresponds to the data show in Figure 2B in the main text. Environmental autocorrelation is denoted as φ whilst environmental variance is denoted as σ^2.

Model type	Model number	Model	DF	AIC	Selection
A priori	1	$DB \sim \sigma^2 + \varphi + \sigma^2 * \varphi$	5	-925.2545	
	2	$DB \sim \sigma^2 + \varphi + \sigma^2 * \varphi + \varphi^2$	6	-953.4019	
	3	$DB \sim \sigma^2 + \varphi + \sigma^2 * \varphi + \varphi^2 + \varphi^3$	7	-980.3957	
	4	$DB \sim \sigma^2 + \varphi + \sigma^2 * \varphi + (\sigma^2)^2$	6	-927.4461	
	5	$DB \sim \sigma^2 + \varphi + \sigma^2 * \varphi + (\sigma^2)^2 + \varphi^2$	7	-956.2009	
	6	$DB \sim \sigma^2 + \varphi + \sigma^2 * \varphi + (\sigma^2)^2 + \varphi^2 + \varphi^3$	8	-983.8628	**SELECTED**
	7	$DB \sim \sigma^2 + \varphi + \sigma^2 * \varphi + (\sigma^2)^2 + (\sigma^2)^3$	7	-925.4511	
	8	$DB \sim \sigma^2 + \varphi + \sigma^2 * \varphi + (\sigma^2)^2 + (\sigma^2)^3 + \varphi^2$	8	-954.2067	
	9	$DB \sim \sigma^2 + \varphi + \sigma^2 * \varphi + (\sigma^2)^2 + (\sigma^2)^3 + \varphi^2 + \varphi^3$	9	-981.8694	
Complete formula		$DB \sim -0.26329 + 1.224845 * [\sigma^2] - 0.027656 * [\varphi] + 0.059247 * [\sigma^2 * \varphi] - 1.215454 * [(\sigma^2)^2] + 0.049503 * [\varphi^2] - 0.1.6741 * [\varphi^3]$			

Supplementary Table 6. Model selection to quantify the effects of environmental autocorrelation and variance on demographic buffering (DB) in *Heliconia tortuosa*. This model selection corresponds to the data show in Figure 2C in the main text. Environmental autocorrelation is denoted as φ whilst environmental variance is denoted as σ^2.

Model type	Model number	Model	DF	AIC	Selection
A priori	1	$DB \sim \sigma^2 + \varphi + \sigma^2 * \varphi$	5	-1891.832	
	2	$DB \sim \sigma^2 + \varphi + \sigma^2 * \varphi + \varphi^2$	6	-1913.937	
	3	$DB \sim \sigma^2 + \varphi + \sigma^2 * \varphi + \varphi^2 + \varphi^3$	7	-1930.921	
	4	$DB \sim \sigma^2 + \varphi + \sigma^2 * \varphi + (\sigma^2)^2$	6	-1895.561	
	5	$DB \sim \sigma^2 + \varphi + \sigma^2 * \varphi + (\sigma^2)^2 + \varphi^2$	7	-1918.323	
	6	$DB \sim \sigma^2 + \varphi + \sigma^2 * \varphi + (\sigma^2)^2 + \varphi^2 + \varphi^3$	8	-1935.878	**SELECTED**
	7	$DB \sim \sigma^2 + \varphi + \sigma^2 * \varphi + (\sigma^2)^2 + (\sigma^2)^3$	7	-1893.568	
	8	$DB \sim \sigma^2 + \varphi + \sigma^2 * \varphi + (\sigma^2)^2 + (\sigma^2)^3 + \varphi^2$	8	-1916.330	
	9	$DB \sim \sigma^2 + \varphi + \sigma^2 * \varphi + (\sigma^2)^2 + (\sigma^2)^3 + \varphi^2 + \varphi^3$	9	-1933.886	
Complete formula		$DB \sim -0.05349 + 0.1897 * [\sigma^2] + [6.797e-04] * [\varphi] - [9.386e-05] * [\sigma^2 * \varphi] - 0.1656 * [(\sigma^2)^2] + 0.005201 * [\varphi^2] - 0.01033 * [\varphi^3]$			

Supplementary Table 7. Model selection to quantify the effects of environmental autocorrelation and variance on the differences of $\sum E_{a_{ij}}^{\sigma^2}$ and $\sum E_{a_{ij}}^{\sigma^2}|$ASD in *Berberis thunbergii*. This model selection corresponds to the data show in Figure 3B in the main text. Environmental autocorrelation is denoted as φ whilst environmental variance is denoted as σ^2.

Model type	Model number	Model	DF	AIC	Selection
A priori	1	$differences{\sim}\sigma^2+\varphi+\sigma^2*\varphi$	5	-741.7773	
	2	$differences{\sim}\sigma^2+\varphi+\sigma^2*\varphi+\varphi^2$	6	-769.6791	
	3	$differences{\sim}\sigma^2+\varphi+\sigma^2*\varphi+\varphi^2+\varphi^3$	7	-890.4373	
	4	$differences{\sim}\sigma^2+\varphi+\sigma^2*\varphi+(\sigma^2)^2$	6	-744.6596	
	5	$differences{\sim}\sigma^2+\varphi+\sigma^2*\varphi+(\sigma^2)^2+\varphi^2$	7	-773.2640	
	6	$differences{\sim}\sigma^2+\varphi+\sigma^2*\varphi+(\sigma^2)^2+\varphi^2+\varphi^3$	8	-898.1634	**SELECTED**
	7	$differences{\sim}\sigma^2+\varphi+\sigma^2*\varphi+(\sigma^2)^2+(\sigma^2)^3$	7	-742.7117	
	8	$differences{\sim}\sigma^2+\varphi+\sigma^2*\varphi+(\sigma^2)^2+(\sigma^2)^3+\varphi^2$	8	-771.3237	
	9	$differences{\sim}\sigma^2+\varphi+\sigma^2*\varphi+(\sigma^2)^2+(\sigma^2)^3+\varphi^2+\varphi^3$	9	-896.2684	
Complete formula		$differences{\sim}-1.969071+3.965287*[\sigma^2]-0.512308*[\varphi]+0.364988*[\sigma^2*\varphi]-1.970633*[(\sigma^2)^2]-0.074138*[\varphi^2]+0.299437*[\varphi^3]$			

Supplementary Table 8. Model selection to quantify the effects of environmental autocorrelation and variance on the differences of $\sum E_{a_{ij}}^{\sigma^2}$ and $\sum E_{a_{ij}}^{\sigma^2}$|ASD in *Calathea crotalifera*. This model selection corresponds to the data show in Figure 3E in the main text. Environmental autocorrelation is denoted as φ whilst environmental variance is denoted as σ^2.

Model type	Model number	Model	DF	AIC	Selection
A priori	1	$differences \sim \sigma^2 + \varphi + \sigma^2 * \varphi$	5	-1718.857	
	2	$differences \sim \sigma^2 + \varphi + \sigma^2 * \varphi + \varphi^2$	6	-1756.217	
	3	$differences \sim \sigma^2 + \varphi + \sigma^2 * \varphi + \varphi^2 + \varphi^3$	7	-1784.586	
	4	$differences \sim \sigma^2 + \varphi + \sigma^2 * \varphi + (\sigma^2)^2$	6	-1759.530	
	5	$differences \sim \sigma^2 + \varphi + \sigma^2 * \varphi + (\sigma^2)^2 + \varphi^2$	7	-1806.057	
	6	$differences \sim \sigma^2 + \varphi + \sigma^2 * \varphi + (\sigma^2)^2 + \varphi^2 + \varphi^3$	8	-1843.012	**SELECTED**
	7	$differences \sim \sigma^2 + \varphi + \sigma^2 * \varphi + (\sigma^2)^2 + (\sigma^2)^3$	7	-1757.962	
	8	$differences \sim \sigma^2 + \varphi + \sigma^2 * \varphi + (\sigma^2)^2 + (\sigma^2)^3 + \varphi^2$	8	-1804.593	
	9	$differences \sim \sigma^2 + \varphi + \sigma^2 * \varphi + (\sigma^2)^2 + (\sigma^2)^3 + \varphi^2 + \varphi^3$	9	-1841.650	
Complete formula		$differences \sim 0.638800 - 1.276380 * [\sigma^2] - 0.019216 * [\varphi] + 0.029041 * [\sigma^2 * \varphi] + 0.637492 * [(\sigma^2)^2] - 0.009601 * [\varphi^2] - 0.018321 * [\varphi^3]$			

Supplementary Table 9. Model selection to quantify the effects of environmental autocorrelation and variance on the differences of $\sum E_{a_{ij}}^{\sigma^2}$ and $\sum E_{a_{ij}}^{\sigma^2}|\text{ASD}$ in *Heliconia tortuosa*. This model selection corresponds to the data show in Figure 3H in the main text. Environmental autocorrelation is denoted as φ whilst environmental variance is denoted as σ^2.

Model type	Model number	Model	DF	AIC	Selection
A priori	1	$differences \sim \sigma^2 + \varphi + \sigma^2 * \varphi$	5	-1380.828	
	2	$differences \sim \sigma^2 + \varphi + \sigma^2 * \varphi + \varphi^2$	6	-1386.508	
	3	$differences \sim \sigma^2 + \varphi + \sigma^2 * \varphi + \varphi^2 + \varphi^3$	7	-1473.794	
	4	$differences \sim \sigma^2 + \varphi + \sigma^2 * \varphi + (\sigma^2)^2$	6	-1392.269	
	5	$differences \sim \sigma^2 + \varphi + \sigma^2 * \varphi + (\sigma^2)^2 + \varphi^2$	7	-1398.431	
	6	$differences \sim \sigma^2 + \varphi + \sigma^2 * \varphi + (\sigma^2)^2 + \varphi^2 + \varphi^3$	8	-1492.824	**SELECTED**
	7	$differences \sim \sigma^2 + \varphi + \sigma^2 * \varphi + (\sigma^2)^2 + (\sigma^2)^3$	7	-1391.522	
	8	$differences \sim \sigma^2 + \varphi + \sigma^2 * \varphi + (\sigma^2)^2 + (\sigma^2)^3 + \varphi^2$	8	-1397.730	
	9	$differences \sim \sigma^2 + \varphi + \sigma^2 * \varphi + (\sigma^2)^2 + (\sigma^2)^3 + \varphi^2 + \varphi^3$	9	-1492.822	
Complete formula		$differences \sim -0.804339 + 1.592423 * [\sigma^2] - 0.017227 * [\varphi] - 0.049087 * [\sigma^2 * \varphi] - 0.782833 * [(\sigma^2)^2] - 0.009305 * [\varphi^2] + 0.067106 * [\varphi^3]$			

Supplementary Table 10. Model selection to quantify the effects of environmental autocorrelation and variance on the mean buffered size in *Berberis thunbergii*. This model selection corresponds to the data show in Figure 3C in the main text. Environmental autocorrelation is denoted as φ whilst environmental variance is denoted as σ^2.

Model type	Model number	Model	DF	AIC	Selection
A priori	1	$mean\ buffered\ size \sim \sigma^2 + \varphi + \sigma^2 * \varphi$	5	-1808.928	
	2	$mean\ buffered\ size \sim \sigma^2 + \varphi + \sigma^2 * \varphi + \varphi^2$	6	-2170.820	
	3	$mean\ buffered\ size \sim \sigma^2 + \varphi + \sigma^2 * \varphi + \varphi^2 + \varphi^3$	7	-2291.923	**SELECTED**
	4	$mean\ buffered\ size \sim \sigma^2 + \varphi + \sigma^2 * \varphi + (\sigma^2)^2$	6	-1807.061	
	5	$mean\ buffered\ size \sim \sigma^2 + \varphi + \sigma^2 * \varphi + (\sigma^2)^2 + \varphi^2$	7	-2169.493	
	6	$mean\ buffered\ size \sim \sigma^2 + \varphi + \sigma^2 * \varphi + (\sigma^2)^2 + \varphi^2 + \varphi^3$	8	-2291.086	
	7	$mean\ buffered\ size \sim \sigma^2 + \varphi + \sigma^2 * \varphi + (\sigma^2)^2 + (\sigma^2)^3$	7	-1805.061	
	8	$mean\ buffered\ size \sim \sigma^2 + \varphi + \sigma^2 * \varphi + (\sigma^2)^2 + (\sigma^2)^3 + \varphi^2$	8	-2167.493	
	9	$mean\ buffered\ size \sim \sigma^2 + \varphi + \sigma^2 * \varphi + (\sigma^2)^2 + (\sigma^2)^3 + \varphi^2 + \varphi^3$	9	-2289.087	
Complete formula		$mean\ buffered\ size \sim 0.6679336 - 0.0313268 * [\sigma^2] - 0.0051673 * [\varphi] - 0.0019560 * [\sigma^2 * \varphi] - 0.0175639 * [\varphi^2] + 0.0133206 * [\varphi^3]$			

Supplementary Table 11. Model selection to quantify the effects of environmental autocorrelation and variance on the mean buffered size in *Calathea crotalifera*. This model selection corresponds to the data show in Figure 3F in the main text. Environmental autocorrelation is denoted as φ whilst environmental variance is denoted as σ^2.

Model type	Model number	Model	DF	AIC	Selection
A priori	1	$mean\ buffered\ size \sim \sigma^2 + \varphi + \sigma^2 * \varphi$	5	-3226.940	
	2	$mean\ buffered\ size \sim \sigma^2 + \varphi + \sigma^2 * \varphi + \varphi^2$	6	-3241.007	
	3	$mean\ buffered\ size \sim \sigma^2 + \varphi + \sigma^2 * \varphi + \varphi^2 + \varphi^3$	7	-3261.367	**SELECTED**
	4	$mean\ buffered\ size \sim \sigma^2 + \varphi + \sigma^2 * \varphi + (\sigma^2)^2$	6	-3225.148	
	5	$mean\ buffered\ size \sim \sigma^2 + \varphi + \sigma^2 * \varphi + (\sigma^2)^2 + \varphi^2$	7	-3239.230	
	6	$mean\ buffered\ size \sim \sigma^2 + \varphi + \sigma^2 * \varphi + (\sigma^2)^2 + \varphi^2 + \varphi^3$	8	-3259.614	
	7	$mean\ buffered\ size \sim \sigma^2 + \varphi + \sigma^2 * \varphi + (\sigma^2)^2 + (\sigma^2)^3$	7	-3223.159	
	8	$mean\ buffered\ size \sim \sigma^2 + \varphi + \sigma^2 * \varphi + (\sigma^2)^2 + (\sigma^2)^3 + \varphi^2$	8	-3237.243	
	9	$mean\ buffered\ size \sim \sigma^2 + \varphi + \sigma^2 * \varphi + (\sigma^2)^2 + (\sigma^2)^3 + \varphi^2 + \varphi^3$	9	-3257.628	
Complete formula		$mean\ buffered\ size \sim 0.8956762 + 0.0007139 * [\sigma^2] + 0.0009980 * [\varphi] - 0.0011847 * [\sigma^2 * \varphi] + 0.0002204 * [\varphi^2] + 0.0005852 * [\varphi^3]$			

Supplementary Table 12. Model selection to quantify the effects of environmental autocorrelation and variance on the mean buffered size in *Heliconia tortuosa*. This model selection corresponds to the data show in Figure 3I in the main text. Environmental autocorrelation is denoted as φ whilst environmental variance is denoted as σ^2.

Model type	Model number	Model	DF	AIC	Selection
A priori	1	$mean\ buffered\ size \sim \sigma^2 + \varphi + \sigma^2 * \varphi$	5	-1971.458	
	2	$mean\ buffered\ size \sim \sigma^2 + \varphi + \sigma^2 * \varphi + \varphi^2$	6	-2090.884	
	3	$mean\ buffered\ size \sim \sigma^2 + \varphi + \sigma^2 * \varphi + \varphi^2 + \varphi^3$	7	-2119.626	**SELECTED**
	4	$mean\ buffered\ size \sim \sigma^2 + \varphi + \sigma^2 * \varphi + (\sigma^2)^2$	6	-1970.073	
	5	$mean\ buffered\ size \sim \sigma^2 + \varphi + \sigma^2 * \varphi + (\sigma^2)^2 + \varphi^2$	7	-2089.940	
	6	$mean\ buffered\ size \sim \sigma^2 + \varphi + \sigma^2 * \varphi + (\sigma^2)^2 + \varphi^2 + \varphi^3$	8	-2118.838	
	7	$mean\ buffered\ size \sim \sigma^2 + \varphi + \sigma^2 * \varphi + (\sigma^2)^2 + (\sigma^2)^3$	7	-1968.075	
	8	$mean\ buffered\ size \sim \sigma^2 + \varphi + \sigma^2 * \varphi + (\sigma^2)^2 + (\sigma^2)^3 + \varphi^2$	8	-2087.944	
	9	$mean\ buffered\ size \sim \sigma^2 + \varphi + \sigma^2 * \varphi + (\sigma^2)^2 + (\sigma^2)^3 + \varphi^2 + \varphi^3$	9	-2116.842	
Complete formula		$mean\ buffered\ size \sim 0.8411429 + 0.0082338 * [\sigma^2] - 0.1178307 * [\varphi] + 0.0912694 * [\sigma^2 * \varphi] + 0.0088286 * [\varphi^2] + 0.0087587 * [\varphi^3]$			

Supplementary Table 13. Model selection to quantify the effects of environmental autocorrelation and variance on the **P-F** contribution in *Berberis thunbergii*. This model selection corresponds to the data show in Figure 4B in the main text. Environmental autocorrelation is denoted as φ whilst environmental variance is denoted as σ^2.

Model type	Model number	Model	DF	AIC	Selection
A priori	1	$\mathbf{P-F}\ contribution \sim \sigma^2 + \varphi + \sigma^2 * \varphi$	5	-8282.140	
	2	$\mathbf{P-F}\ contribution \sim \sigma^2 + \varphi + \sigma^2 * \varphi + \varphi^2$	6	-8747.482	
	3	$\mathbf{P-F}\ contribution \sim \sigma^2 + \varphi + \sigma^2 * \varphi + \varphi^2 + \varphi^3$	7	-8783.395	
	4	$\mathbf{P-F}\ contribution \sim \sigma^2 + \varphi + \sigma^2 * \varphi + (\sigma^2)^2$	6	-8286.741	
	5	$\mathbf{P-F}\ contribution \sim \sigma^2 + \varphi + \sigma^2 * \varphi + (\sigma^2)^2 + \varphi^2$	7	-8804.515	
	6	$\mathbf{P-F}\ contribution \sim \sigma^2 + \varphi + \sigma^2 * \varphi + (\sigma^2)^2 + \varphi^2 + \varphi^3$	8	-8853.171	**SELECTED**
	7	$\mathbf{P-F}\ contribution \sim \sigma^2 + \varphi + \sigma^2 * \varphi + (\sigma^2)^2 + (\sigma^2)^3$	7	-8284.763	
	8	$\mathbf{P-F}\ contribution \sim \sigma^2 + \varphi + \sigma^2 * \varphi + (\sigma^2)^2 + (\sigma^2)^3 + \varphi^2$	8	-8802.731	
	9	$\mathbf{P-F}\ contribution \sim \sigma^2 + \varphi + \sigma^2 * \varphi + (\sigma^2)^2 + (\sigma^2)^3 + \varphi^2 + \varphi^3$	9	-8851.442	
Complete formula		$\mathbf{P-F}\ contribution \sim [5.943e-08] - [1.541e-07] * [\sigma^2] + [1.951e-09] * [\varphi] - [6.017e-09] * [\sigma^2 * \varphi] + [1.208e-07] * [(\sigma^2)^2] + [1.038e-08] * [\varphi^2] + [3.633e-09] * [\varphi^3]$			

Supplementary Table 14. Model selection to quantify the effects of environmental autocorrelation and variance on the **P-F** contribution in *Calathea crotalifera*. This model selection corresponds to the data show in Figure 4C in the main text. Environmental autocorrelation is denoted as φ whilst environmental variance is denoted as σ^2.

Model type	Model number	Model	DF	AIC	Selection
A priori	1	$\mathbf{P-F}\ contribution \sim \sigma^2 + \varphi + \sigma^2 * \varphi$	5	-8011.995	
	2	$\mathbf{P-F}\ contribution \sim \sigma^2 + \varphi + \sigma^2 * \varphi + \varphi^2$	6	-8040.051	
	3	$\mathbf{P-F}\ contribution \sim \sigma^2 + \varphi + \sigma^2 * \varphi + \varphi^2 + \varphi^3$	7	-8067.034	
	4	$\mathbf{P-F}\ contribution \sim \sigma^2 + \varphi + \sigma^2 * \varphi + (\sigma^2)^2$	6	-8014.190	
	5	$\mathbf{P-F}\ contribution \sim \sigma^2 + \varphi + \sigma^2 * \varphi + (\sigma^2)^2 + \varphi^2$	7	-8042.851	
	6	$\mathbf{P-F}\ contribution \sim \sigma^2 + \varphi + \sigma^2 * \varphi + (\sigma^2)^2 + \varphi^2 + \varphi^3$	8	-8070.503	**SELECTED**
	7	$\mathbf{P-F}\ contribution \sim \sigma^2 + \varphi + \sigma^2 * \varphi + (\sigma^2)^2 + (\sigma^2)^3$	7	-8012.195	
	8	$\mathbf{P-F}\ contribution \sim \sigma^2 + \varphi + \sigma^2 * \varphi + (\sigma^2)^2 + (\sigma^2)^3 + \varphi^2$	8	-8040.857	
	9	$\mathbf{P-F}\ contribution \sim \sigma^2 + \varphi + \sigma^2 * \varphi + (\sigma^2)^2 + (\sigma^2)^3 + \varphi^2 + \varphi^3$	9	-8068.509	
Complete formula		$\mathbf{P-F}\ contribution \sim -[3.810e-08] + [1.773e-07] * [\sigma^2] - [4.000e-09] * [\varphi] + [8.580e-09] * [\sigma^2 * \varphi] - [1.760-07] * [(\sigma^2)^2] + [7.155e-09] * [\varphi^2] - [1.545e-08] * [\varphi^3]$			

Supplementary Table 15. Model selection to quantify the effects of environmental autocorrelation and variance on the **P-F** contribution in *Heliconia tortuosa*. This model selection corresponds to the data show in Figure 4D in the main text. Environmental autocorrelation is denoted as φ whilst environmental variance is denoted as σ^2.

Model type	Model number	Model	DF	AIC	Selection
A priori	1	$\mathbf{P-F}\ contribution \sim \sigma^2 + \varphi + \sigma^2 * \varphi$	5	-8958.788	
	2	$\mathbf{P-F}\ contribution \sim \sigma^2 + \varphi + \sigma^2 * \varphi + \varphi^2$	6	-8986.418	
	3	$\mathbf{P-F}\ contribution \sim \sigma^2 + \varphi + \sigma^2 * \varphi + \varphi^2 + \varphi^3$	7	-9010.538	
	4	$\mathbf{P-F}\ contribution \sim \sigma^2 + \varphi + \sigma^2 * \varphi + (\sigma^2)^2$	6	-8962.007	
	5	$\mathbf{P-F}\ contribution \sim \sigma^2 + \varphi + \sigma^2 * \varphi + (\sigma^2)^2 + \varphi^2$	7	-8990.381	
	6	$\mathbf{P-F}\ contribution \sim \sigma^2 + \varphi + \sigma^2 * \varphi + (\sigma^2)^2 + \varphi^2 + \varphi^3$	8	-9015.246	**SELECTED**
	7	$\mathbf{P-F}\ contribution \sim \sigma^2 + \varphi + \sigma^2 * \varphi + (\sigma^2)^2 + (\sigma^2)^3$	7	-8960.023	
	8	$\mathbf{P-F}\ contribution \sim \sigma^2 + \varphi + \sigma^2 * \varphi + (\sigma^2)^2 + (\sigma^2)^3 + \varphi^2$	8	-8988.399	
	9	$\mathbf{P-F}\ contribution \sim \sigma^2 + \varphi + \sigma^2 * \varphi + (\sigma^2)^2 + (\sigma^2)^3 + \varphi^2 + \varphi^3$	9	-9013.267	
Complete formula		$\mathbf{P-F}\ contribution \sim -[6.997e-09] + [2.657e-08] * [\sigma^2] - [4.225e-11] * [\varphi] + [7.592e-10] * [\sigma^2 * \varphi] - [2.392e-08] * [(\sigma^2)^2] + [8.669e-10] * [\varphi^2] - [1.796e-09] * [\varphi^3]$			
